# Golgi-targeting viscosity probe for the diagnosis of Alzheimer’s disease

**DOI:** 10.1038/s41598-023-50789-8

**Published:** 2024-01-16

**Authors:** Wenjing Wu, Lingyu Zhao, Yuanyuan Zhang, Jinchao Wei, Juanjuan Han, Yangyang Zhang, Zhenwen Zhao

**Affiliations:** 1 Beijing National Laboratory for Molecular Sciences, CAS Research/Education Center for Excellence in Molecular Sciences, Key Laboratory of Analytical Chemistry for Living Biosystems, Beijing Mass Spectrum Center, Institute of Chemistry, Chinese Academy of Sciences, Beijing, 100190 China; 2 University of Chinese Academy of Sciences, Beijing, 100049 China

**Keywords:** Analytical chemistry, Biomarkers

## Abstract

Early diagnosis and intervention of Alzheimer’s disease (AD) are particularly important to delay the pathological progression. Although fluorescent probes have been widely employed for investigating and diagnosing AD, their biological applications are significantly restricted due to the low penetration ability of the blood–brain barrier (BBB) in vivo. In this study, we reported the first Golgi-targeted two-photon (TP) fluorescent probe, **DCM-DH,** for detecting viscosity in the Golgi apparatus. The probe was rationally designed to exhibit superior analytical performance including high sensitivity, specific Golgi-targeting, efficient BBB penetration ability, and deep tissue penetration (247 μm) in the brains of AD model mice. Using the probe, we demonstrated that the fluorescence intensity in the human liver cancer cell (HepG2 cells) was higher than that of human normal liver cell (LO2 cells), and the brain viscosity of AD model mice increased significantly. We anticipate that this competent tool could be easily extended to other AD biomarkers for fundamental research on this detrimental disease.

## Introduction

Cellular and subcellular viscosity plays a critical role in various cell processes, including the transportation of chemical signals and the diffusion of biomolecules^1–3^. Abnormal deviations in cellular viscosity have been implicated in a range of diseases, such as hypertension, atherosclerosis, and Alzheimer’s^4,5^. The changes in cellular viscosity are closely linked to the subcellular microenvironment^6^. For this reason, monitoring viscosity fluctuations at the subcellular organelle level can provide valuable insights not only into the biological chemical processes but also into the pathogenesis and diagnosis of diseases^7^.

The Golgi apparatus is the central hub of the exocytic and endocytic channels in intracellular membrane transport^8^. It is responsible for sorting newly synthesized and recycled proteins, as well as lipids, for subsequent transport to the cell surface, secretory granules, or the endosomal system^9^. However, when the Golgi apparatus malfunctions, proteins and lipids are unable to be sorted properly and may accumulate, leading to changes in Golgi viscosity^10^. Given that the pathogenesis of Alzheimer’s disease (AD) is closely linked to oxidative stress, the misfolding of proteins such as amyloid-*β* (A*β*) plaques and the hyperphosphorylation of tau protein in neurons, Golgi viscosity can be considered as a vital biomarker for AD and a potential indicator for early diagnosis^11^. Therefore, it is imperative to develop effective tools for visualizing and measuring Golgi viscosity.

Fluorescence imaging has become widely used for locating and dynamically monitoring bioactive species and important factors related to the cell microenvironment due to its high sensitivity, great spatiotemporal resolution, and non-destructive nature. These factors include hydrogen peroxide (H_2_O_2_), polarity, and viscosity, among others^12–14^. Two-photon (TP) fluorescence imaging has emerged as a promising tool for deep tissue and in vivo imaging thanks to its ability to integrate little photodamage, deep tissue penetration depth, and low background fluorescence interference^15–18^. Several viscosity-sensitive fluorescent probes have been developed based on molecular rotors^12,19–22^. However, these probes lack subcellular targeting ability and cannot detect viscosity in the Golgi^23,24^. In addition, early diagnosis of AD in vivo requires the probe that can penetrate the blood–brain barrier (BBB)^25^. Another concern in designing viscosity-sensitive fluorescent probes is to avoid biological autofluorescence^26,27^. Therefore, TP fluorescent probes, when combined with advanced TP fluorescence microscopy, can penetrate deeper into tissues and have lower optical damage while providing higher resolution compared to one-photon fluorescent probes^28,29^. To address these limitations, we have developed a Golgi viscosity-activatable TP fluorescent probe (**DCM-DH**) that can non-invasively localize AD lesions-induced viscosity changes.

## Experimental section

### Materials and instruments

Imidazole, methyl 4-hydroxycinnamate, tert-butyl dimethylsiyl chloride (TBSCl), diisobutyl aluminium hydride (DIBAL-H), MnO_2_, tetrabutyl ammonium fluoride (TBAF), *N,N*-dimethylformamide (DMF), ethyl acetate, acetic acid (AcOH), 1-(2-hydroxyphenyl) ethenone, sulfuric acid, Serine, Citric acid, Cysteine (Cys), dichloromethane, toluene and piperidine were purchased from Energy Chemical (Shanghai, China). Malononitrile, Homocysteine (Hcy), trans-Cinnamaldehyde, carboxylesterase (CE), Glucose oxidase (GOx), Vitamin C (VC) and bovine serum albumin (BSA) were obtained from J&K Scientific Ltd. (Beijing, China). *N*-acetyl-l-cysteine (NAC), Catalase, Trypsin and Glutamine were purchased from Aladdin Biochemical Technology Co., Ltd. (Shanghai, China). amyloid-*β*42 (A*β*42) was obtained from Ziyu Biotechnology Co., Ltd. (Shanghai, China). Reactive oxygen species were prepared according to the previous report^1^. Golgi-Tracker Green, MitoTracker Green, ER-Tracker Green, LysoTracker Green, Hoechst 33342 and BODIPY 493/503 were purchase from Beyotime Biothechnology (Shanghai, China) and Thermo Fisher Scientific. 3-(4,5-Dimethylthiazol-2-yl)-2,5-diphenyltetrazolium bromide (MTT) was acquired from Serva Electrophoresis GmbH (Heidelberg, Germany). High-glucose Dulbecco’s modified eagle medium (DMEM), penicillin–streptomycin and fetal bovine serum (FBS) were purchased from Thermo Fisher Scientific Co. Ltd (MA, USA). LO2 (human hepatocytes) cells and SH-SY5Y (human neuroblastoma cell) cells used in this paper were purchased from Kaiji Biotechnology Co., Ltd (Jiangsu, China). Female BALB/c mice (age: 4–5 weeks, weight: 16–19 g), wild-type C57 and AD female mice (age: five-month-old, weight: 24–28 g) were acquired from SPF Biotechnology Co., Ltd. (Beijing, China).

^1^H and ^13^C NMR spectra were measured by Bruker Avance III HD 400 spectrometer. High-resolution mass spectra (HRMS) were obtained by a Bruker SolariX mass spectrometer equipped with a 9.4 T superconducting magnet. Viscosity values were recorded by a Brookfield rotational viscometer. Ultraviolet–visible (UV–vis) absorption spectra were recorded on a TU-1900 UV–vis double-beam spectrometer (Purkinje General, China) in 1 cm quartz cells. Fluorescence spectra were performed on a F-4600 fluorescence spectrophotometer (Hitachi, Tokyo Japan) in 1 cm × 1 cm quartz cells. Fresh brain tissue was cut into sections by microtome cryostat (Leica CM1950, Germany). Confocal fluorescence images were recorded on an FV 1200-IX83 confocal laser scanning microscope and image processing was made with Olympus software FV10-ASW.

### Synthesis and characterization of DCM-DH and DCM-PH

The (E)-3-(4-hydroxyphenyl)acrylaldehyde (DH), **DCM** and **DCM-OH** were prepared following the previous methods and their synthetic route were shown in the Fig. S1^2–4^. **DCM** (0.21 g, 1.0 mmol) and DH (0.15 g, 1.0 mmol) were dissolved in toluene (20 mL) with piperidine (0.5 mL) and acetic acid (0.5 mL) under nitrogen protection. The resulting mixture was refluxed for 24 h. Then the mixed liquid was cooled to room temperature and extracted twice with ethyl acetate. Anhydrous sodium sulfate was used to dry the organic layer. After the solvent was evaporated under reduced pressure, the residue was purified by silica-gel chromatography with CH_2_Cl_2_/CH_3_OH (v/v, 50:1) as the eluent, affording **DCM-DH** as a crimson solid (193 mg, yield: 57%). ^1^H, ^13^C NMR and ESI-FTICR mass spectra of **DCM-DH** were shown in Figs. S2–S4. ^1^H NMR (400 MHz, DMSO-*d*_*6*_): δ 9.92 (s, 1H), 8.69 (dd, *J* = 9.0, 3.8 Hz, 1H), 7.94–7.84 (m, 1H), 7.72 (dd, *J* = 8.6, 4.3 Hz, 1H), 7.61–7.49 (m, 2H), 7.48–7.41 (m, 2H), 7.09–6.91 (m, 2H), 6.85 (d, *J* = 5.7 Hz, 1H), 6.80 (d, *J* = 8.3 Hz, 2H), 6.71 (dd, *J* = 15.2, 4.9 Hz, 1H). ^13^C NMR (75 MHz, DMSO-*d*_*6*_) δ 159.0, 158.4, 152.5, 151.9, 141.2, 140.7, 135.2, 129.3, 127.2, 126.0, 124.5, 124.3, 120.4, 118.9, 117.3, 117.0, 115.9, 105.7, 59.3. MALDI-FTICR MS: *m/z* Calcd. for C_22_H_13_N_2_O_2_^–^: 337.09715 [M-H]^–^; found: *m/z* 337.09817.

**DCM** (0.21 g, 1.0 mmol) and trans-cinnamaldehyde (PH) (0.13 g, 1.0 mmol) were dissolved in toluene (20 mL) with piperidine (0.5 mL) and acetic acid (0.5 mL) under nitrogen protection. The compound **DCM-PH** was produced according to the **DCM-DH** procedure (219 mg, 68%).^1^H, ^13^C NMR and ESI-FTICR mass spectra of **DCM-PH** were shown in Figs. S5–S7. ^1^H NMR (400 MHz, DMSO-*d*_*6*_) δ 8.70 (d, *J* = 8.4 Hz, 1H), 7.91 (t, *J* = 7.8 Hz, 1H), 7.75 (d, *J* = 8.5 Hz, 1H), 7.58 (dd, *J* = 16.1, 8.9 Hz, 4H), 7.41 (t, *J* = 7.4 Hz, 2H), 7.38–7.31 (m, 1H), 7.25–7.10 (m, 2H), 6.92 (s, 1H), 6.85 (d, *J* = 15.2 Hz, 1H). ^13^C NMR (75 MHz, DMSO-*d*_*6*_) δ 157.9, 152.7, 151.9, 140.3, 139.7, 136.0, 135.4, 129.2, 128.9, 127.5, 127.3, 126.1, 124.6, 122.6, 119.0, 117.2, 117.0, 115.8, 106.3, 60.1. ESI-FTICR MS: *m/z* Calcd. for C_22_H_14_N_2_O: 322.11006 [M]; found: *m/z* 322.11131.

### General details for fluorescence and viscosity measurements

The stock solution of **DCM-DH**, **DCM-OH** and **DCM-PH** (10 mM) was dissolved in DMSO. The solutions of probes (final concentration 10 μM) with different viscosity were obtained by adding the stock solution of probes to the mixture (3 mL) of water-glycerol mixture with different volume proportions. The solutions were shaken constantly for 1 h and their fluorescence were performed. The viscosity of each solution is listed in Table S1. The solutions to the various biologically related species were prepared with ultrapure water.

### Measurement of two-photon cross-section

The two-photon cross-section of **DCM-DH** were measured in DMSO/PBS buffer (v/v, 3/7, 100 μM) as described in literature^5^. The cross-sections of **DCM-DH** were calculated by using the following formula: δ = δr × (Fs × фr × nr)/(Fr × фs × ns), where δ, F, ф and n were TP cross-section, spectral integral area, quantum yield and concentration, respectively; s and r stood for sample and reference, respectively. Fluorescein (aqueous, 10 μM, pH 11) was used as a reference probe. From 735 to 900 nm, **DCM-DH** was calculated to have a two-photon absorption cross-section of δ_max_ = 55.3 GM (1 GM = 10^–50^ cm^4^ s/photon molecule) at 830 nm.

### Cell culture and cytotoxicity assay

SH-SY5Y and LO2 cells were grown in T75 glass culture flask in high-glucose Dulbecco’s modified eagle medium (DMEM) supplemented with 10% (v/v) FBS and 1% (v/v) penicillin–streptomycin at 37 °C in a humidified 5% CO_2_ incubator.

To ensure the safety and suitability of **DCM-DH** for biological applications, the cytotoxicity of the designed probe **DCM-DH** to SH-SY5Y and LO2 cells was evaluated by the standard MTT assay. SH-SY5Y and LO2 cells were seeded into 96-well cell culture plates at a density of 8000 cells/well for 24 h before use. Then, the medium was replaced by the solutions of **DCM-DH** at concentrations of 5, 10, 20, 30, 40 and 50 μM in 100 μL of the medium and the cells were cultured for another 12 h. Afterwards, 10 μL of MTT solution (5 mg/mL) was added to each well. After 4 h, the medium was removed and 150 μL of DMSO was added to dissolve the formazan. The absorption at 570 nm was recorded using microplate reader (Molecular Devices SpectraMax i3 BioTek, USA) and the percentage of cell viability was calculated: cell viability (%) = (mean of absorbance value of the treatment group/mean of absorbance value of control) × 100.

### Confocal fluorescence imaging of living cells

The cells were seeded on 35 mm confocal dishes and incubated overnight. For subcellular localization experiment, cells were incubated with **DCM-DH** (10 μM) for 30 min and then incubated with each corresponding commercial organelle-specific dye for 15 min. The cell culture medium of each group was removed, and all cells were washed with PBS three times before fluorescence imaging. The fluorescence of **DCM-DH** was recorded with excitation of 488 nm and emission of 600–700 nm. To image the Golgi stress, monensin (10 μM) were add to the culture medium and incubated with cells for 30 min. Afterwards, the culture medium was replaced with fresh medium and the cells were incubated with **DCM-DH** (10 μM) or DCFH-DA (10 μM) for 30 min. To investigate intracellular ROS clearance, the cells were pretreated with NAC (10 μM) for 1 h and then cultured with monensin (10 μM) for 30 min and **DCM-DH** (10 μM) or DCFH-DA (10 μM) for 30 min. To establish the Alzheimer’s disease model, SH-SY5Y cells were pretreated with A*β*42 (10 μM) for 24 h and then stained with **DCM-DH** (10 μM) for 30 min before confocal imaging.

### HPLC analyses

High performance liquid chromatography (HPLC) analyses were performed to monitor the reaction process. A solution of 100 μL of mouse serum was added to the mixture containing **DCM-DH** (1 μL, 1 mM in DMSO) and 900 μL of PBS (0.2 M, pH: 7.4) in a final volume of 1 mL. The mixture was incubated for 10 h at 37 °C. 1 mL **DCM-DH** (1 µM in PBS containing 0.1% DMSO) and the mixture then analyzed on a Shimadzu LC-40 XR preparative HPLC system (Shimadzu, Japan) for detection. The measurement parameters were set as follows. Column: Dikma Bio-Bond column (150 × 2.1 mm), the mobile phase A was H_2_O containing 0.1% TFA, mobile phase B was acetonitrile containing 0.1% TFA. The HPLC separations were 30 min/sample and the gradient were as follows: (1) 0 min, 10% B; (2) 30 min, 100% B. (All the changes were linear, flow rate: 0.2 mL/min, detection wavelength: 450 nm).

### Mouse brain imaging

Wild-type (WT) and AD-model mouse brains were cut into 5 μm slices using microtome cryostat (Leica CM1950, Germany). Slices were incubated with different commercial organelle trackers, including Golgi apparatus Tracker Green (Golgi tracker), Lysosome Tracker Green (Lyso. tracker), Endoplasmic Reticulum Tracker Green (ER tracker), Mitochondria Tracker Green (Mito. tracker), Lipid Droplets Tracker (BODIPY 493/503, LD tracker) and Hoechst 33342 (Nuc. tracker) for 30 min, and then incubated with **DCM-DH** (10 μM) for 30 min, then imaged after washing by PBS.

### Ethics approval and consent to participate

All animal procedures were performed in accordance with the Guidelines for the Care and Use of Laboratory Animals and approved by the Institutional Animal Care and Use Committee (IACUC) of Peking University (approval protocol no. 2018-0033). The study is reported in accordance with ARRIVE guidelines (https://arriveguidelines.org).

### BBB penetration test and in vivo fluorescence imaging

Mice were injected intravenously (*i.v.*) with **DCM-OH** (0.4 mg/kg, 20% DMSO and 80% 1,2-propylene glycol, 50 μL) or **DCM-DH** (0.4 mg/kg, 20% DMSO and 80% 1,2-propylene glycol, 50 μL). Mice injected with PBS were used as negative control. Mice were euthanized 30 min post injections, and the organs of each different group were collected for fluorescence imaging and subsequent hematoxylin and eosin (H&E) staining. For animal euthanasia, mice were euthanized by CO_2_ asphyxiation. The death of animal was verified by cervical dislocation.

### One or two-photon fluorescence imaging

One-Photon fluorescence images were recorded on an FV 1200-IX83 confocal laser scanning microscope (PerkinElmer, USA) with excitation filter of 488 nm as well as emission filter of 600 nm. Two-photon fluorescence images were detected on an A1R MP confocal laser scanning microscope (Nikon, Japan) with 820 nm femtosecond-pulsed wavelength excitation and 600–700 nm emission. During the confocal experiment, all parameters were remained constant.

## Results

### Design and synthesis of DCM-DH

**Figure 1:**
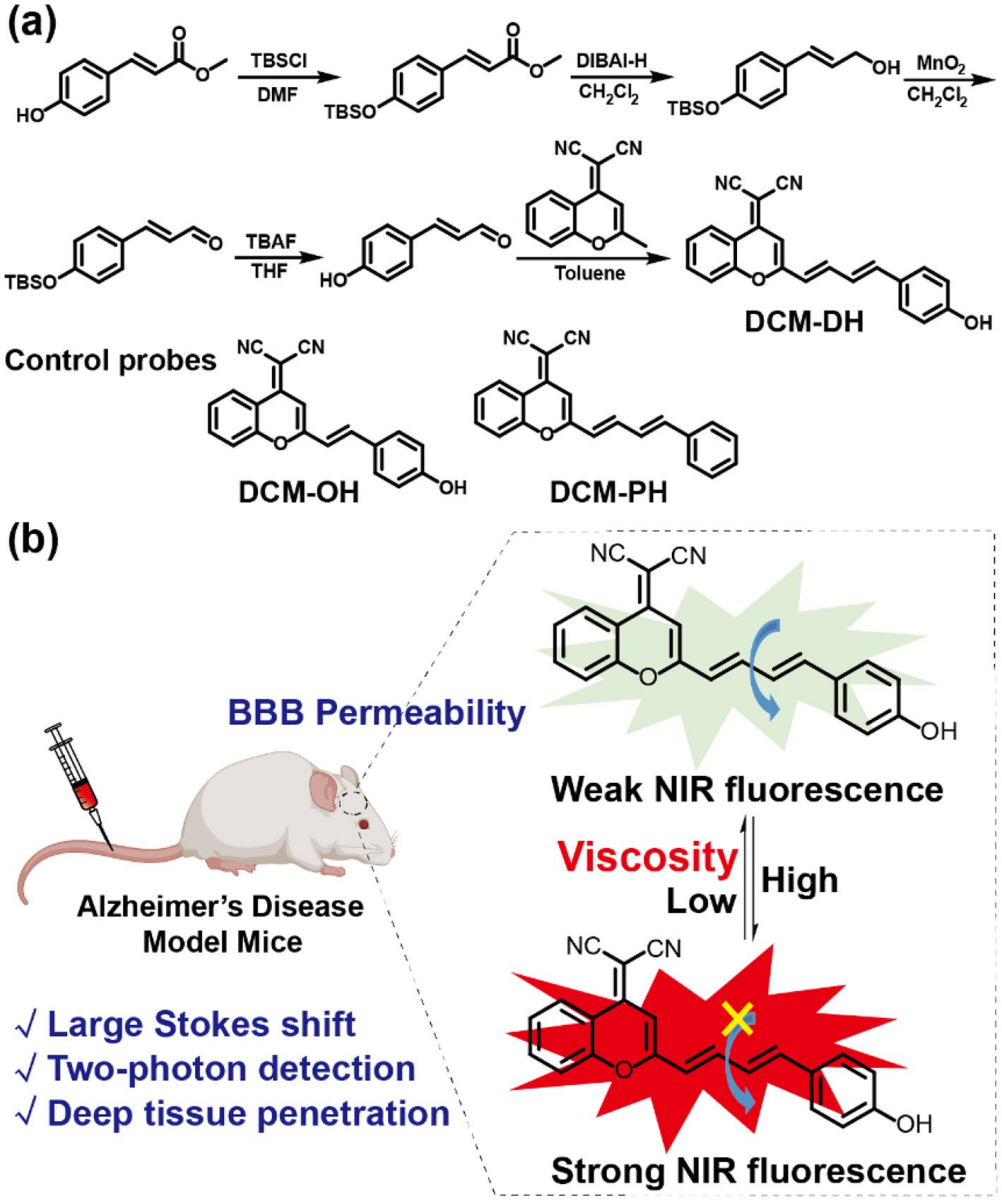
(**a**) Synthesis and structure of **DCM-DH**. Structure of control probes. (**b**) Schematic illustration of viscosity-responsive and blood–brain barrier (BBB) permeable probe **DCM-DH**.

In our previous work, a DCM-based fluorescent probe was developed for live cell and xenograft tumour tissue imaging of NQO1 (NAD(P)H quinone oxidoreductase-1)^30^. To achieve a rapid and sensitive response to viscosity changes, we extended the conjugated links (carbon–carbon double bond) of **DCM-OH** (Fig. 1a). The resulting **DCM-DH** has a molecular rotor structure with two small, symmetric π-conjugations, in which the 4H-chromene (electron acceptor) and phenol moiety (electron donor) are connected by two rotatable vinyl bonds to form a D-π-A structure. As ambient viscosity increases, intramolecular rotation is restricted, inhibiting non-radiative decay of the excited state and resulting in fluorescence enhancement of **DCM-DH**. The probe demonstrated excellent targeting ability toward the Golgi apparatus and high selectivity for detecting viscosity changes. Using the probe, we identified a significant increase in viscosity in the Golgi apparatus in a cellular AD model. Most importantly, the probe can cross the BBB and enable TP imaging in mouse brain tissues (Fig. 1b), providing a powerful chemical tool for monitoring Golgi apparatus viscosity function in AD.

To the best of our knowledge, Golgi apparatus targeted viscosity-activated TP probes have not been proposed for diseases diagnosis, anti-diseases drug efficacy evaluation, or revealing the mechanism of diseases development in vivo. Therefore, we designed three push–pull phenylpolyenylchromene fluorophores (**DCM-DH**, **DCM-PH** and **DCM-OH**) differentiated by length of the conjugated links or push–pull electronic ability (Fig. S1). We adopted the hydroxylphenylpolyenyl group for its good electron-donating and transporting capabilities and utilized the 4H-chromene group to enlarge the molecule conjugate chain, thereby shifting the emission spectrum to the red region. Additionally, we introduced the malononitrile moiety for its good electron-withdrawing ability and Golgi apparatus targeting ability. We expected that the rotor of the probes would freely rotate in lower viscosity media, such as in normal tissue, resulting in rapid consumption of energy through nonradiative relaxation pathways and weaker fluorescence (Fig. 1b). Conversely, with an increase in medium viscosity in the Golgi apparatus, the probe’s rotor would become more restricted, leading to a decrease in energy dissipation through nonradiative pathways and stronger fluorescence (Fig. 1b).

Specifically, the Golgi-targeted fluorophore, **DCM-DH**, was synthesized by conjugating the 2-(2-methyl-4H-chromen-4-ylidene) malononitrile with 4-hydroxyphenylacrylaldehyde in the presence of piperidine. The synthesis procedures of **DCM-DH**, along with its control compounds **DCM-PH** and **DCM-OH** were presented in Supporting Information Fig. S1. All intermediates and target compounds were fully characterized using ^1^H and ^13^C NMR spectroscopy, as well as mass spectrometry. Supplementary information (Figs. S2–S7) demonstrated the results of these analyses. Notably, the MALDI-FTICR mass spectrum of **DCM-DH** exhibited a peak at m/z = 337.09817 (Fig. S4), which corresponded to [M-H]^-^.

### Spectroscopic properties and sensitivity to viscosity

**Figure 2:**
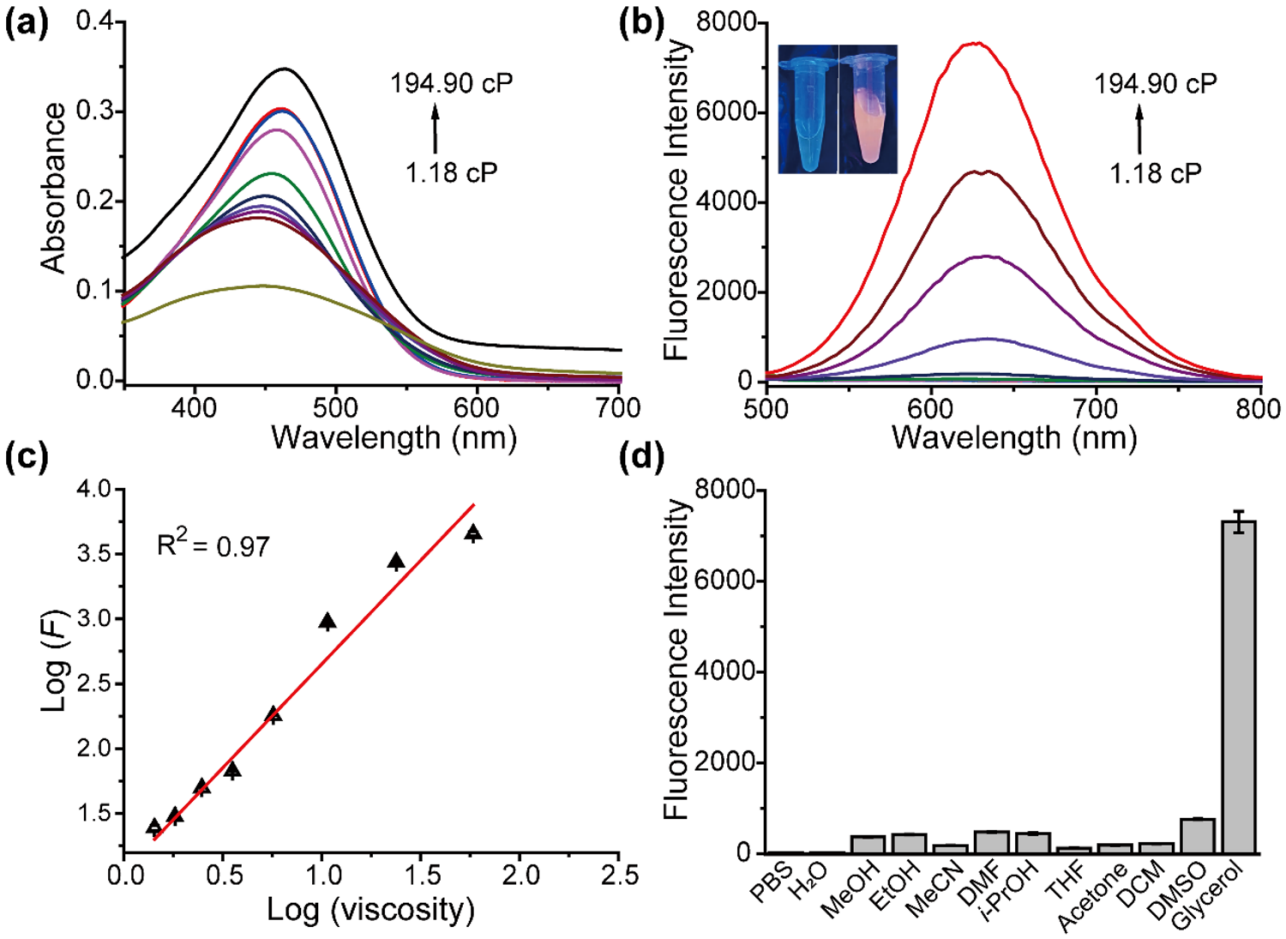
The viscosity responsive properties of the **DCM-DH** probe. (**a**) Absorption spectra and (**b**) Fluorescent spectra of **DCM-DH** (10 μM) in glycerol-water mixed system with different viscosities. (**c**) The liner curve of fluorescence intensity (*F*_650_) against various viscosity (*η*). (**d**) Fluorescence behavior of **DCM-DH** (10 μM) in different solvents (PBS, H_2_O, MeOH, EtOH, MeCN, DMF, *i*-PrOH, THF, Acetone, CH_2_Cl_2_, DMSO, 90% glycerol). λ_ex_/_em_ = 480/640 nm.

In order to characterize the spectroscopic properties of **DCM-DH**, **DCM-PH** and **DCM-OH**, we conducted an investigation into their spectral response to viscosity in a water-glycerol system (Table S1). The UV–vis and photoluminescence spectrometer was used. In low-viscosity media, **DCM-DH** showed an absorption band at approximately 480 nm and weak fluorescent at 640 nm when excited at 480 nm, which was consistent with its rotatability (Fig. 2a, b). In comparison to **DCM-PH**, **DCM-OH** and **DCM-DH** exhibited a significant red-shift in UV absorption and fluorescence emission due to increased conjugation and the electron-donating effect of hydroxyl (Fig. 2a, b and Figs. S8, S9). As the viscosity of the medium was increased from 1.18 to 194.90 cP, **DCM-PH** did not exhibit any observable fluorescence enhancement (Table S1 and Fig. S9). However, the fluorescence of **DCM-OH** and **DCM-DH** increased dramatically (Fig. 2b and Figs. S9, S10). **DCM-DH** exhibited higher fluorescence intensity in response to viscosity compared to **DCM-OH** (about 8000 vs 5000), making it more sensitive. Furthermore, **DCM-DH**’s longer emission wavelength (640 nm) and larger Stokes shift (160 nm) due to the extension of the conjugated bridge enabled high-performance imaging in living mice. Based on the viscosity titration studies, we observed a good linear relationship (R^2^ = 0.97, Fig. 2c) between the maximum emission intensity (log *F*) and the viscosity of the medium (log *η*) calculated by Förster-Hoffmann equation^31^. Using fluorescence titrations, we found the limit of detection (LOD) of **DCM-DH** towards viscosity was calculated to be 0.026 cP, which was nearly an order of magnitude higher than other probes^32^. The fluorescence lifetime of **DCM-DH** exhibited a similar tendency toward viscosity (Fig. S11), which gradually prolonged as the increase of the viscosity.

To verify the specificity of **DCM-DH** for viscosity, we conducted experiments using various biologically related species, including reactive oxygen species (ROS), reactive nitrogen species (RNS), and proteins. Figures S12 and S13 demonstrated that only an increase in viscosity resulted in a significant increase in the fluorescence signal at 640 nm, without interference from other ions, ROS, RNS, or biomacromolecules. Particularly, we confirmed that the fluorescence intensity of **DCM-DH** was unaffected by different solvents (Fig. 2d) and BSA protein (the most abundant protein in serum) at physiological concentrations under different viscosities (Fig. S13). These findings suggested that **DCM-DH** had high specificity for viscosity detection. To ensure accurate viscosity detection in brain, we also investigated the potential interference of A*β* aggregates on **DCM-DH**. We incubated DCM-DH with A*β* aggregates, and the fluorescence properties increased by less than 5% (Fig. S14), indicating that A*β* aggregates had a weak effect on **DCM-DH** probe. Additionally, it was widely known that the Golgi apparatus was a weakly acidic organelle, with a physiological pH ranging from 6.0 to 6.7^23^. To assess the feasibility of **DCM-DH**’s applications in biological conditions, we tested PBS buffer with different pH values (pH 4.0–11.0) and found no effect on the fluorescence intensity of **DCM-DH** (Fig. S15). This suggested that **DCM-DH** was suitable for use in various biological environments. Furthermore, we determined the maximum TP absorption cross section (*δ*_max_) of **DCM-DH** to be 55.3 GM at 830 nm (Fig. S16), indicating its potential for high-resolution TP imaging. With such a large TP absorption cross section, **DCM-DH** had great potential for use in high-resolution imaging applications.

### Fluorescence imaging of the Golgi viscosity fluctuations

**Figure 3:**
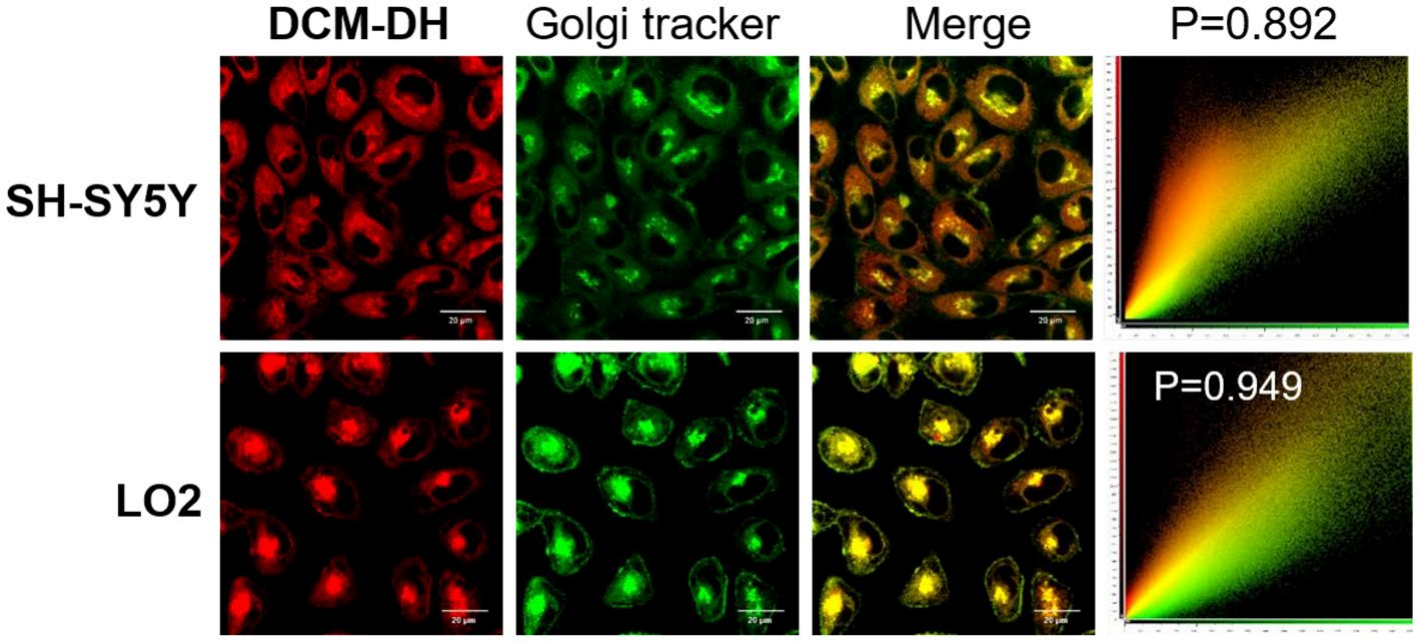
Confocal fluorescence images of **DCM-DH** in SH-SY5Y and LO2 cells. Colocalization images of cells co-incubated with **DCM-DH** (10 μM, excited at 488 nm, collected from 600 to 700 nm) for the red channel and the Golgi tracker for the green channel. Scale bar: 20 μm.

After identifying **DCM-DH** as our preferred viscosity sensitive probe, we investigated its target to the subcellular organ. Firstly, its low cytotoxicity with concentration lower than 50 μM was confirmed (Fig. S17). Secondly, we compared the cell imaging results of three probes and found that **DCM-DH** had the best imaging properties (Fig. S18). Therefore, **DCM-DH** was used in this study. We co-incubated it with different commercial organelle trackers, including Golgi apparatus Tracker Green (Golgi tracker), Lysosome Tracker Green (Lyso. tracker), Endoplasmic Reticulum Tracker Green (ER tracker), Mitochondria Tracker Green (Mito. tracker), Lipid Droplets Tracker (BODIPY 493/503, LD tracker) and Hoechst 33342 (Nuc. tracker) in SH-SY5Y and LO2 cells. As shown in Fig. 3 and Figs. S19–S22, **DCM-DH** and Golgi Tracker demonstrated the best fluorescence overlap with a Pearson’s coefficient of 0.892 and 0.949, respectively, while with other organelle tracker, with Pearson’s coefficient from 0.001 to 0.766. The specific targeting of **DCM-DH** to the Golgi apparatus could be attributed to its lipophilicity and the –CN group, which was an effective Golgi-targeted moiety^33–35^. The lipophilicity of compounds played a critical role in their cell uptake and distribution in subcellular organelles, as reported in previous studies. Therefore, both lipophilicity and the effective Golgi-targeted group enabled **DCM-DH** to accumulate in the Golgi region.

**Figure 4:**
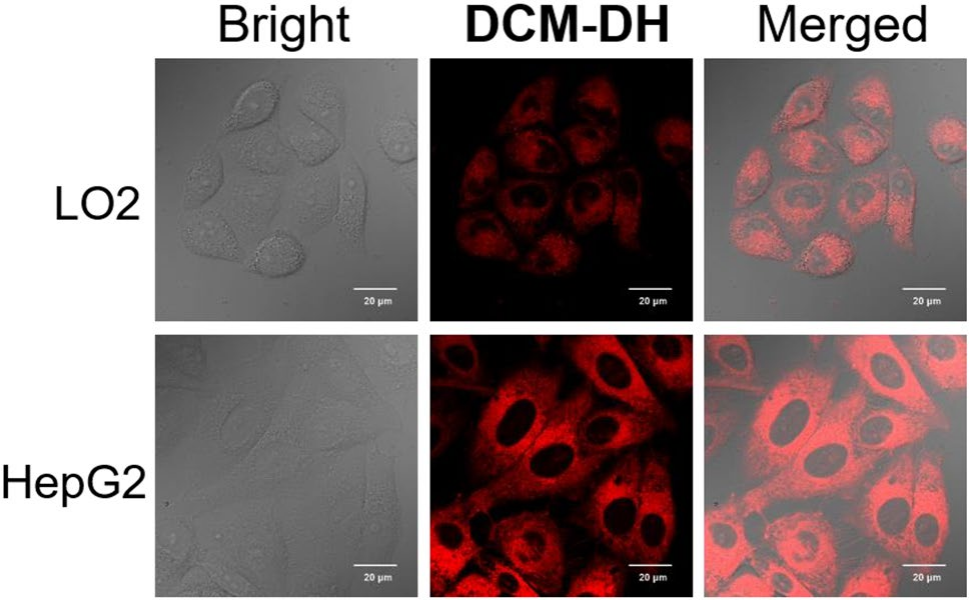
Confocal images of LO2 cells (human normal liver cells) and HepG2 cells (human liver cancer cells) incubated with **DCM-DH** (10 μM) for 30 min. Scale bar: 20 μm.

The tumor microenvironment plays a crucial role in the development and progression of cancer, and has become an important direction for research and the fight against cancer. Viscosity is a key microenvironmental parameter, and because it can control the rate of intracellular diffusion and bimolecular reactions, it will become a potential indicator for cancer diagnosis. We also recorded the mean fluorescence intensity in both the tumor and normal cells (Fig. 4), and observed that the fluorescence intensity in HepG2 cells, a human liver cancer cell line, was higher than that in LO2 cells, a human normal liver cell line. This significant difference confirmed the potential of using **DCM-DH** to diagnose cancer by sensing Golgi viscosity.

### Monitoring the Golgi viscosity during Golgi stress

**Figure 5:**
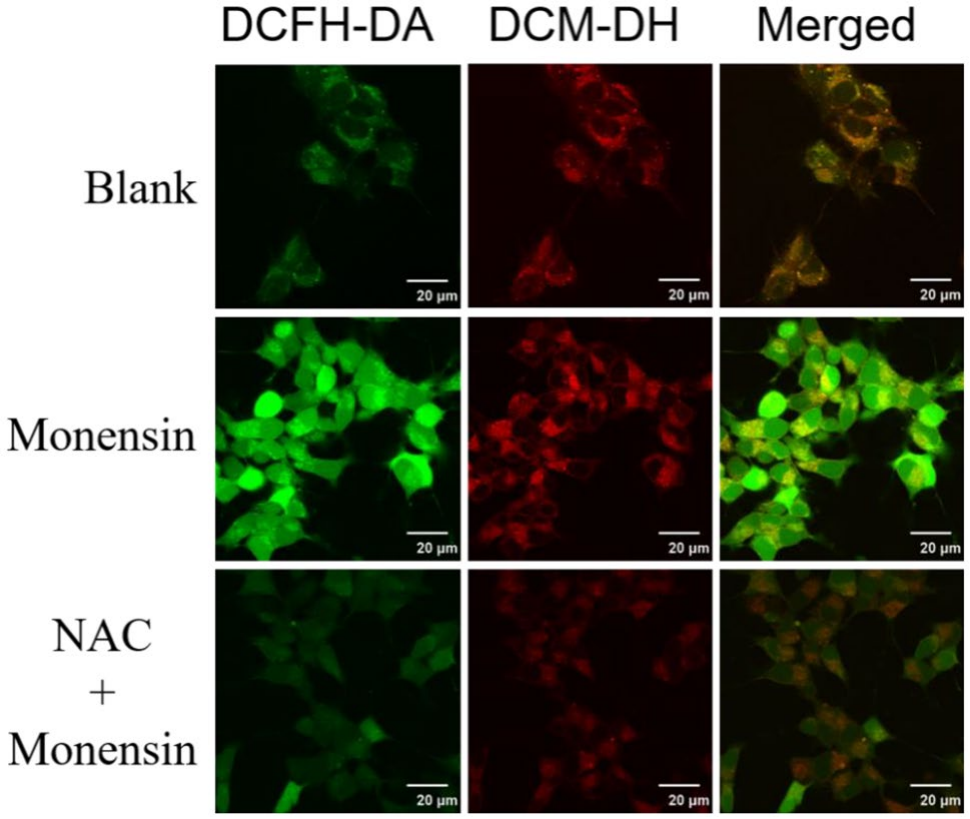
Confocal fluorescence images of intracellular ROS (green channel) and the Golgi viscosity (red channel) in SH-SY5Y cells by DCFH-DA and **DCM-DH**. Blank group: cells were incubated with DCFH-DA (10 μM) and **DCM-DH** (10 μM). The monensin group: cells were pretreated with monensin (10 μM) for 30 min and then incubated with DCFH-DA (10 μM) and **DCM-DH** (10 μM). The NAC + monensin group: cells were pretreated with NAC (10 μM) + monensin (10 μM) for 30 min and then incubated with DCFH-DA (10 μM) and **DCM-DH** (10 μM). Scale bar: 20 μm.

It has been reported that monensin may cause Golgi stress by disrupting cellular ion homeostasis, but the effect of this stress on Golgi viscosity remains unclear^23,36^. To investigate this, we used the **DCM-DH** probe to monitor Golgi viscosity changes in human neuroblastoma cells (SH-SY5Y cells) exposed to monensin. Our results showed that Golgi stress induced by monensin significantly increased Golgi viscosity, as evidenced by enhanced red fluorescence in the Golgi apparatus as shown in Figs. 5 and S23. This suggested that Golgi viscosity could be a useful indicator of Golgi stress.

To understand the underlying mechanism of Golgi viscosity changes during Golgi stress, we studied the role of ROS fluctuations. ROS have been shown to affect cellular viscosity by oxidizing intracellular components^12^. We used 2′,7′-dichlorodihydrofluorescein diacetate (DCFH-DA) to measure ROS levels in control and monensin treated cells, and found that monensin led to an increase in green fluorescence, indicating ROS production. Addition of the antioxidant N-acetylcysteine (NAC) significantly reduced ROS levels. Furthermore, simultaneous monitoring of Golgi viscosity and ROS levels showed that both were increased in monensin treated cells, and addition of NAC reduced both (Figs. 5 and S23, S24). These results suggested that ROS fluctuations are involved in regulating Golgi viscosity during Golgi stress.

### Imaging Golgi viscosity in cellular AD model

**Figure 6:**
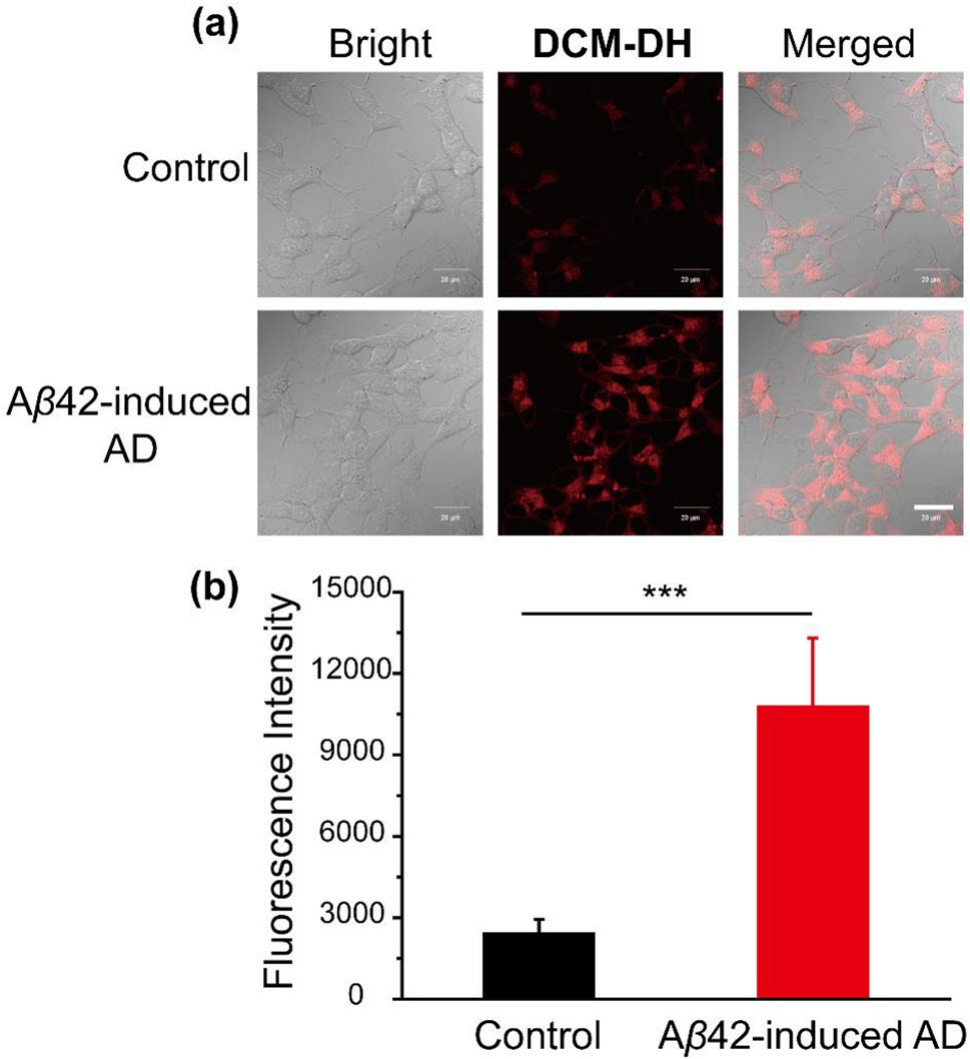
(**a**) Confocal fluorescence images of SH-SY5Y cells with **DCM-DH** in the cellular AD model. Control group: cells were incubated with **DCM-DH** (10 μM). The A*β*42-induced AD group: cells were pretreated with A*β*42 (10 μM) for 24 h and then incubated with **DCM-DH** (10 μM). Scale bar: 20 μm. (**b**) Relative pixel intensity of the corresponding fluorescence images in panels (**a**). Corresponding fluorescence intensity histogram (mean ± SD, n = 3). λ_ex_ = 488 nm, collected 600–700 nm. Significant differences (***P < 0.001) are obtained by Student’s *t*-test.

Building on the exceptional intracellular imaging capabilities of **DCM-DH**, we proceeded to establish an AD model in SH-SY5Y cells using a previously reported method^23^. Specifically, we treated SH-SY5Y cells with A*β*42 for 24 h and subsequently incubated them with **DCM-DH**. The presence of A*β*42 induced Golgi stress and altered Golgi viscosity. Our results, as depicted in Fig. 6a, b, revealed that the fluorescence intensity of the A*β*42-induced AD group was approximately 4.5 times higher than that of the control group, signifying a significant increase in Golgi viscosity in AD cells.

### Fluorescence imaging of DCM-DH in live mice

Alzheimer’s disease, which is the most common form of dementia, has become a global concern^5^. Evidence suggests that the pathogenesis of the disease is linked to the aggregation of hyperphosphorylated tau and A*β* in the brain, which may lead to an increase in viscosity^4^. Since there is no cure for AD, early diagnosis has been proposed as a crucial aspect of AD care. By using fluorescent imaging to visualize the cerebral viscosity, it is possible to monitor the progress of AD based on the physiological differences between normal and pathological mouse brains. However, the low permeability of the blood–brain barrier has been a significant obstacle for early diagnosis of AD in the past.

To address this issue, we investigated the potential of **DCM-DH** for biological imaging in live mice, inspired by its excellent intracellular imaging capability. Our in vitro stability studies showed that **DCM-DH** has high stability in mouse serum, with more than 83% of the intact probe remaining after 10 h of incubation at 37 °C (Fig. S25). We then injected **DCM-DH** into normal mice via the tail vein at a dose of 0.4 mg/kg and measured the fluorescent intensity of major organs, including the brain, heart, liver, spleen, lung, and kidneys, 30 min post-injection using the IVIS.

**Figure 7:**
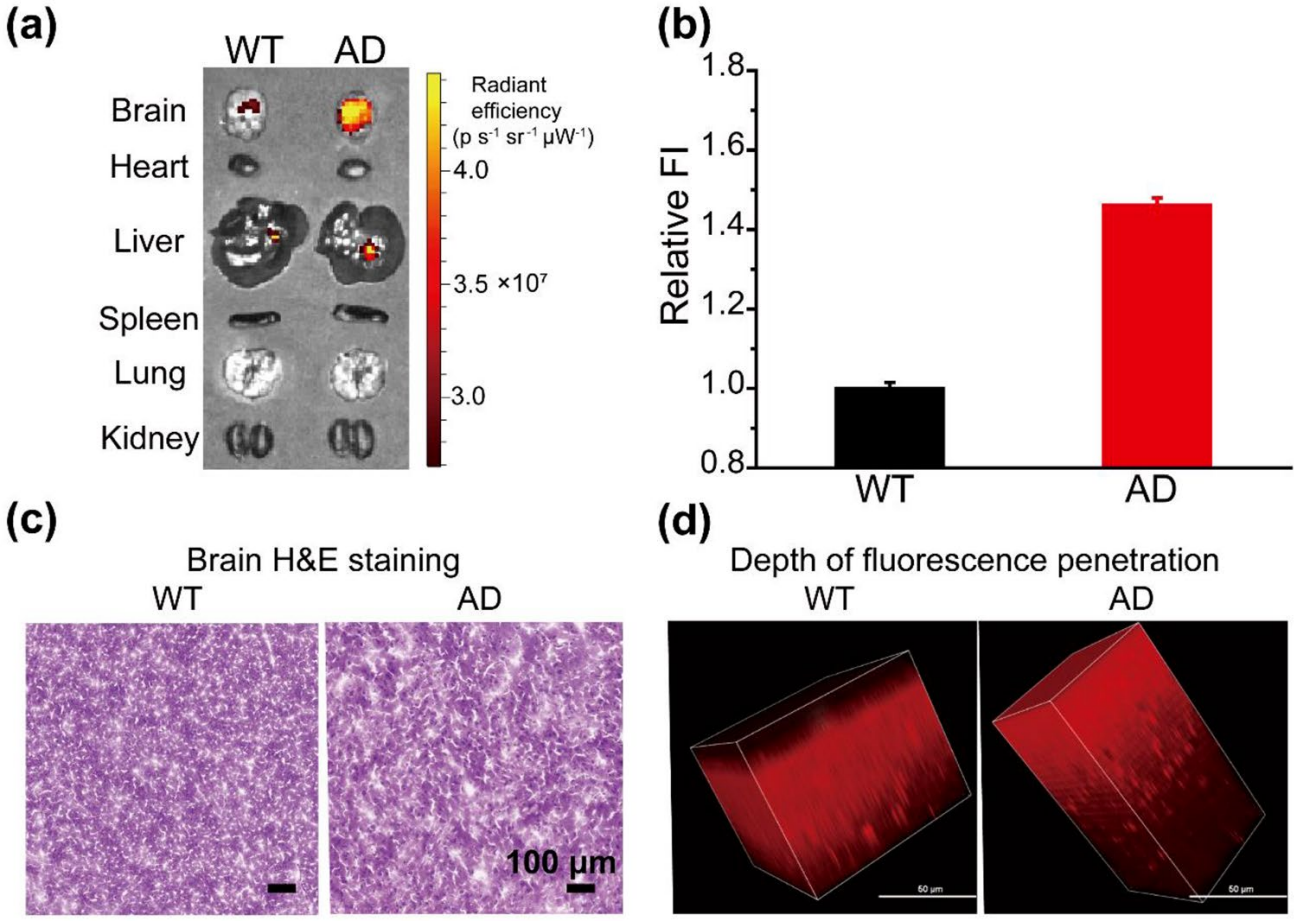
(**a**) The distribution of **DCM-DH** in the main organs of wild-type (WT) and AD-model mice. **DCM-DH** was intravenously injected into the mice, and the mice were sacrificed after 30 min. The main organs were removed for imaging. (**b**) Relative fluorescence intensity in brains of wild-type (WT) and AD mice. (**c**) H&E staining of WT and AD mouse brains. Scale bar: 100 μm. (**d**) The two-photon 3D z-stack fluorescence images of experimental mouse brains upon 30 min intravenously injection with **DCM-DH** (0.4 mg/kg). (λ_ex_ = 820 nm, collected from 600 to 700 nm. Scale bar: 50 μm).

Our results showed that **DCM-DH** could penetrate the blood–brain barrier and accumulate in the brain (Fig. S26). Previous studies have suggested that various key parameters, including molecular weight, lipophilicity, and charge density, can impact the BBB permeability of molecules^37,38^. **DCM-DH** possessed a longer conjugated carbon–carbon double-bond bridge, which enhances its lipophilicity and may improve its ability to penetrate the BBB. Due to **DCM-DH**’s excellent BBB-penetrating capability, we investigated its imaging ability in vivo in an AD model. After injecting **DCM-DH** into normal (wild-type, WT) and AD mice via tail vein, we observed a remarkable increase (about 46%) in fluorescence in the brains of AD mice compared to normal mice (Fig. 7a, b). Currently, the imaging of A*β* aggregates in the brain using fluorescent probes for specific diagnosis of AD is considered a late biomarker. As shown in Table S2, compared to reported A*β* probes, **DCM-DH** has a longer emission wavelength (640 nm), larger stokes shift (160 nm), and lower injected dose (0.4 mg/kg), which enables high-performance imaging of brain viscosity. More importantly, changes in brain viscosity may occur early in AD, making it a valuable tool for early detection of AD-related brain diseases.

We also evaluated the organelle targeting ability of **DCM-DH** at the mouse brain sections, and found that **DCM-DH** had the best fluorescence overlap with the Golgi apparatus with a Pearson’s colocalization coefficient of 0.915, indicating that **DCM-DH** had good performance in detecting the viscosity in Golgi apparatus at the tissue level (Fig. S27).

Additionally, liver function analysis and H&E study of major mouse organs (Fig. 7c and Figs. S28–S30), including the brain, heart, spleen, lung, and kidney, showed that the injection of **DCM-DH** had a minimal negative effect on mouse health, indicating its high biocompatibility for in vivo imaging.

### Two-photon imaging in mouse brains

We incubated **DCM-DH** with wild-type (WT) and AD-model mice brain sections, as depicted in Fig. S31, the results revealed that the fluorescence intensity of the AD-model mice brain sections were higher than that of the control group, signifying a significant increase in Golgi viscosity in the AD mouse brain compared with that in the WT mouse brain. Furthermore, we investigated whether **DCM-DH** could effectively image endogenous viscosity in deep brain tissue using two-photon fluorescence microscopy. In this experiment, we sacrificed WT and AD mice 30 min after tail vein injection of **DCM-DH** and isolated their brains for ex vivo imaging. As depicted in Fig. 7d, the two-photon fluorescence signals of endogenous viscosity were prominently detected in AD mouse brain tissues at depths of up to approximately 247 μm, which exceeded the depth range of most existing probes^28,39^. Conversely, the penetration depth in WT mouse brains was only ~ 92 μm, which corresponded to their comparatively lower viscosity levels (Fig. 7d). These remarkable imaging outcomes demonstrate the significant potential of **DCM-DH** for two-photon deep-tissue imaging and early AD diagnosis due to its exceptional penetrating capacity.

## Discussion

Alzheimer's disease is a neurodegenerative disease characterized by progressive cognitive dysfunction and behavioral impairment^11^. It is one of the important diseases affecting human health in today’s world, causing serious social and economic burden. The development of efficient, sensitive, and accurate diagnostic methods for Alzheimer's disease is of great significance and demand. Up to now, specific diagnosis of AD is mainly focused on imaging of amyloid-*β* (A*β*) aggregates in the brain; however, this is a very late biomarker^5^. In addition, the blood–brain barrier (BBB) prevents most molecules from entering the central nervous system, which limits the applications of fluorescent probes with large molecular frameworks for brain imaging.

In summary, we present a novel fluorescent probe, **DCM-DH**, which exhibits excellent specificity in visualizing viscosity in cells and mice in vivo using one- and two-photon fluorescence spectroscopy. The probe displays highly selective and sensitive fluorescence response to viscosity in the Golgi apparatus over other biologically relevant species. By using the probe, we were able to speculate on the relationship between increased viscosity in the Golgi apparatus and AD, revealing a significant increase of viscosity during the A*β*-induced AD process. Furthermore, **DCM-DH** was found to be capable of effectively crossing the BBB and specific imaging viscosity in AD model mouse brains with excellent deep tissue penetration. With its superb sensitivity, selectivity, and biocompatibility, we anticipate that this viscosity-activated two-photon fluorescent probe holds great promise for the early diagnosis of diseases associated with viscosity, such as AD, as well as for investigating the related efficacy of therapy.

### Supplementary Information

Supplementary Information.

## Data Availability

All data generated and analyzed during this study are included in this published article and its supplementary information files.
