# Supplementary material for: Golgi-targeting viscosity probe for the diagnosis of Alzheimer’s disease
Source: Sci Rep. 2024 Jan 16;14:1336. doi: 10.1038/s41598-023-50789-8 (PMC10791657; doi:10.1038/s41598-023-50789-8)
Supplement: Supplementary file 1 — Supplementary Information. [file 41598_2023_50789_MOESM1_ESM.docx]

Supporting Information for

**Golgi-targeting viscosity probe for the diagnosis of Alzheimer’s disease**

Wenjing Wu^1,2^, Lingyu Zhao^1,2^, Yuanyuan Zhang^1,2^, Jinchao Wei^1,2^, Juanjuan Han^1,2^,

Yangyang Zhang^1,2,^* & Zhenwen Zhao^1,2,^*

^1^Beijing National Laboratory for Molecular Sciences, CAS Research/Education Center for Excellence in Molecular Sciences, Key Laboratory of Analytical Chemistry for Living Biosystems, Beijing Mass Spectrum Center, Institute of Chemistry, Chinese Academy of Sciences, Beijing 100190, China

^2^University of Chinese Academy of Sciences, Beijing 100049, China

Email: zhangyy@iccas.ac.cn (Y. Zhang), zhenwenzhao@iccas.ac.cn (Z. Zhao).

**Figure S1.** Synthesis of the designed probes.


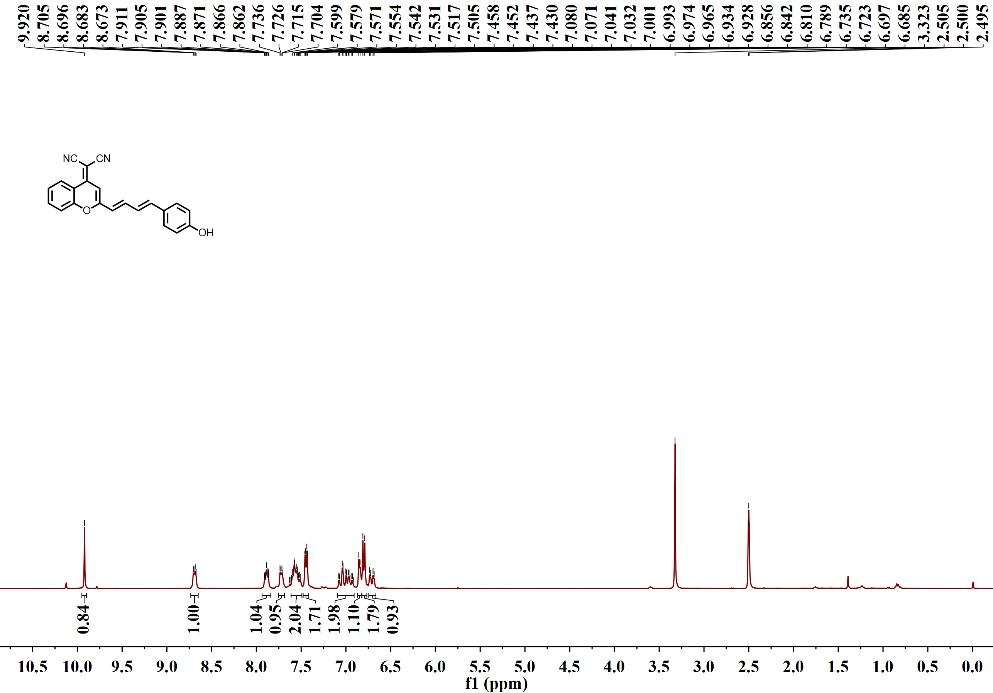


**Figure S2.** ^1^H NMR spectrum of **DCM-DH**. (400 MHz, DMSO-*d_6_*, 298 K).


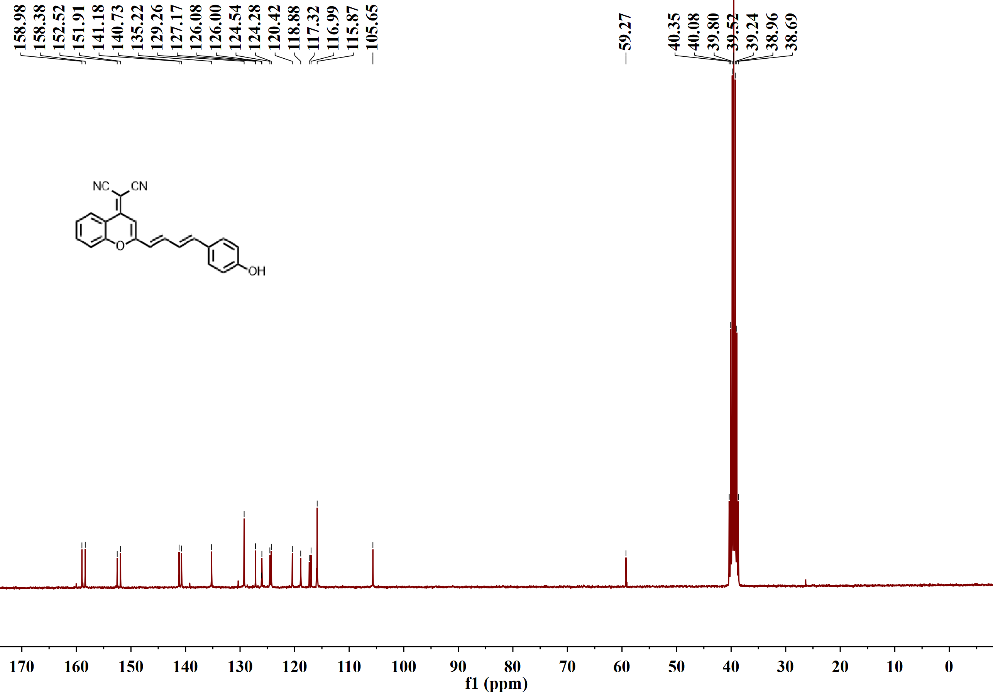


**Figure S3.** ^13^C NMR spectrum of **DCM-DH**. (75 MHz, DMSO-*d_6_*, 298 K).


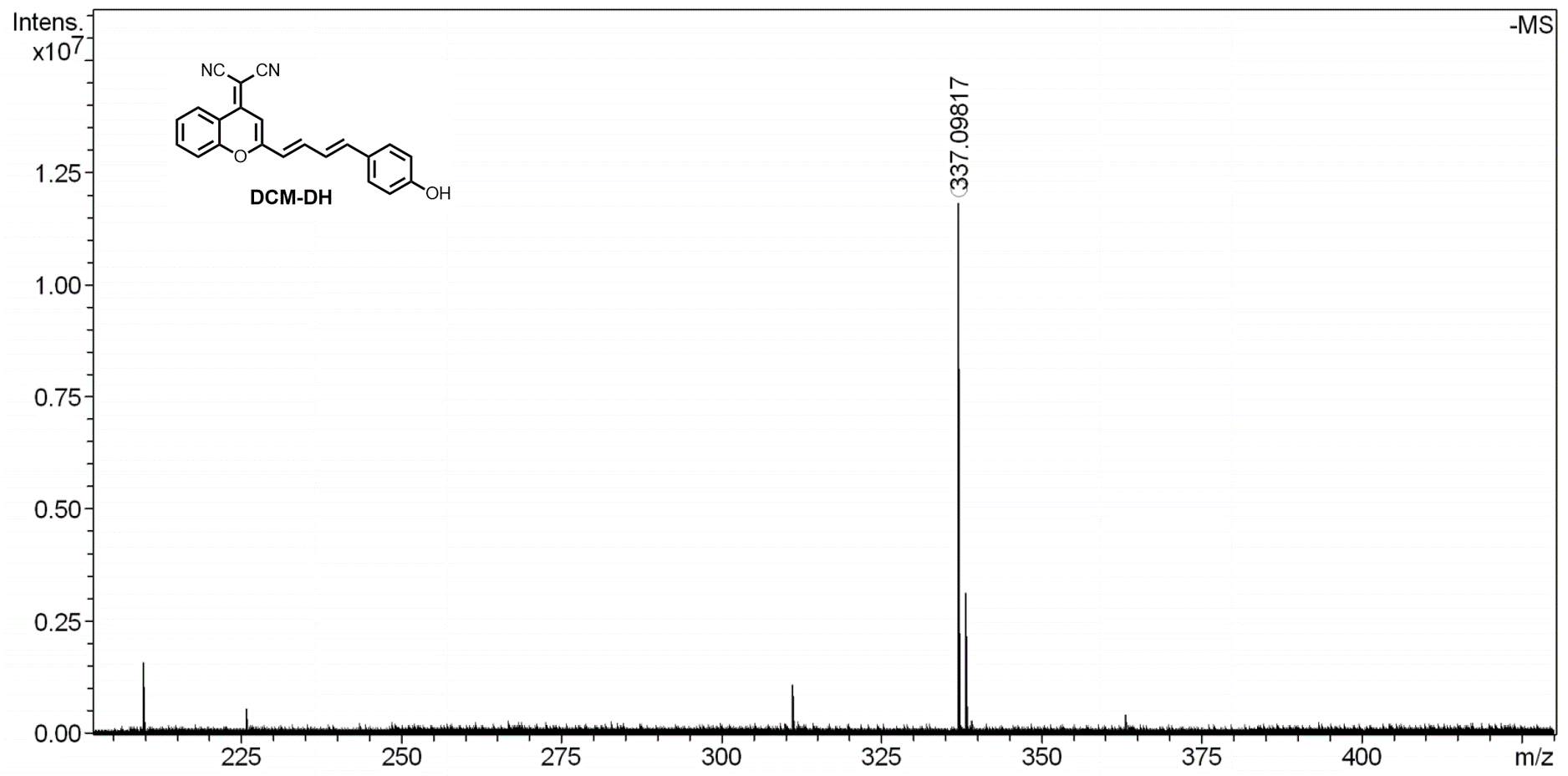


**Figure S4.** MALDI-FTICR MS spectrum of **DCM-DH**.


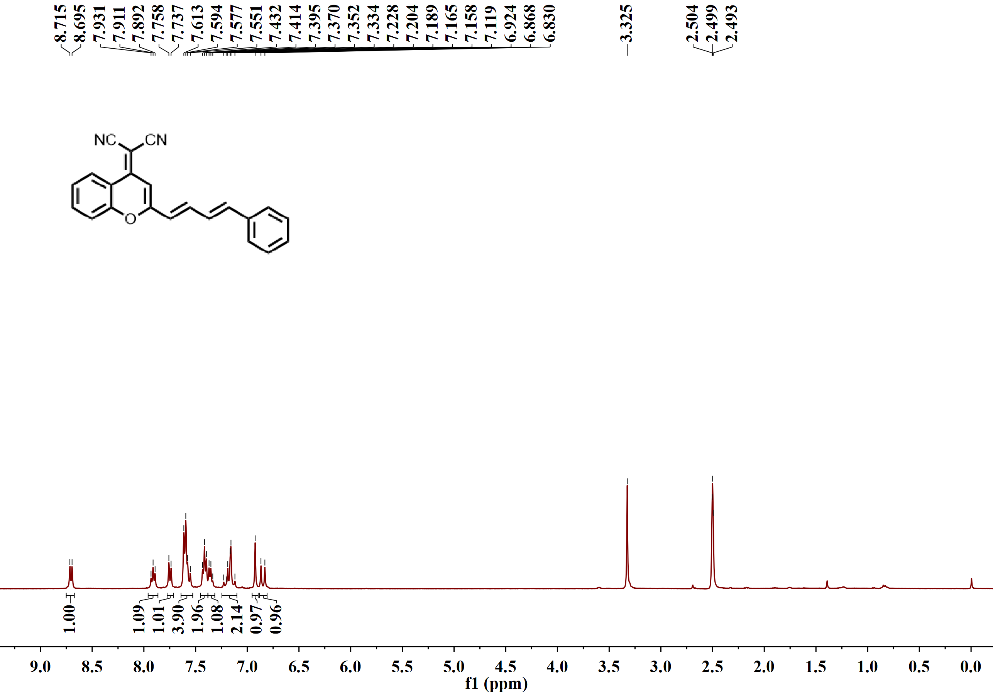


**Figure S5.** ^1^H NMR spectrum of **DCM-PH**. (400 MHz, DMSO-*d_6_*, 298 K).


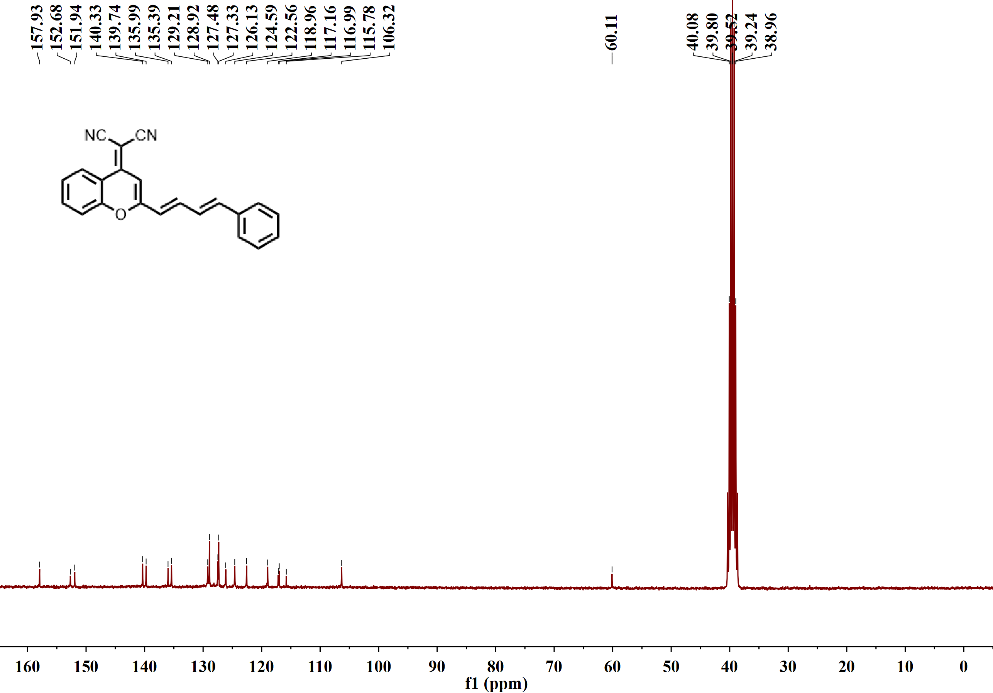


**Figure S6.** ^13^C NMR spectrum of **DCM-PH**. (75 MHz, DMSO-*d_6_*, 298 K).


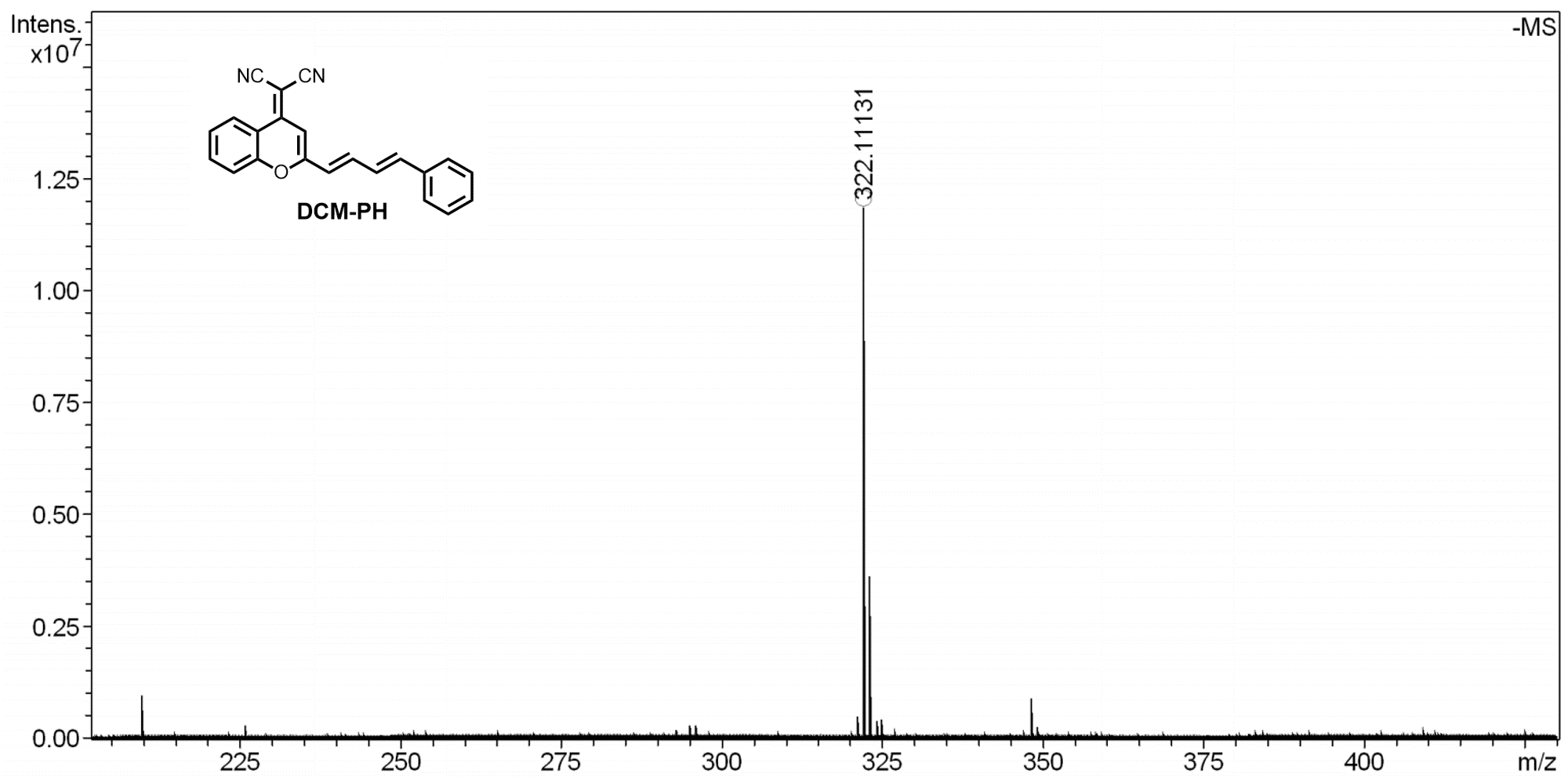


**Figure S7.** MALDI-FTICR MS spectrum of **DCM-PH**.

**Table S1**. The viscosity of the water-glycerol mixture in different proportions.

| water | glycerol | viscosity | water | glycerol | viscosity |
| --- | --- | --- | --- | --- | --- |
| (v%) | (v%) | (cP) | (v%) | (v%) | (cP) |
| 100 | 0 | 1.18 | 50 | 50 | 5.70 |
| 90 | 10 | 1.43 | 40 | 60 | 10.72 |
| 80 | 20 | 1.81 | 30 | 70 | 23.86 |
| 70 | 30 | 2.48 | 20 | 80 | 58.42 |
| 60 | 40 | 3.54 | 10 | 90 | 194.90 |


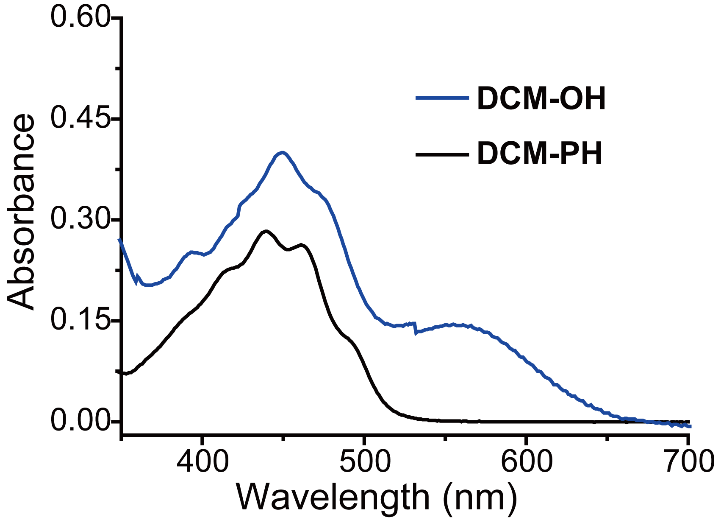


**Figure S8.** UV-vis absorption spectra of **DCM-OH** (10 μM) and **DCM-PH** (10 μM).


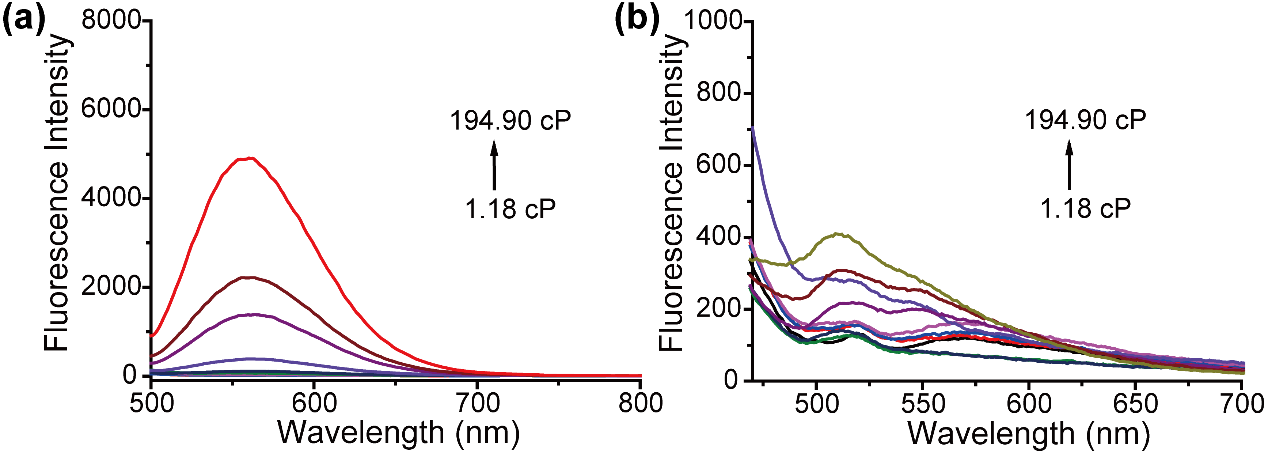


**Figure S9.** Fluorescence spectra of (a) **DCM-OH** (10 μM) and (b) **DCM-PH** (10 μM) in water-glycerol system with different viscosities.


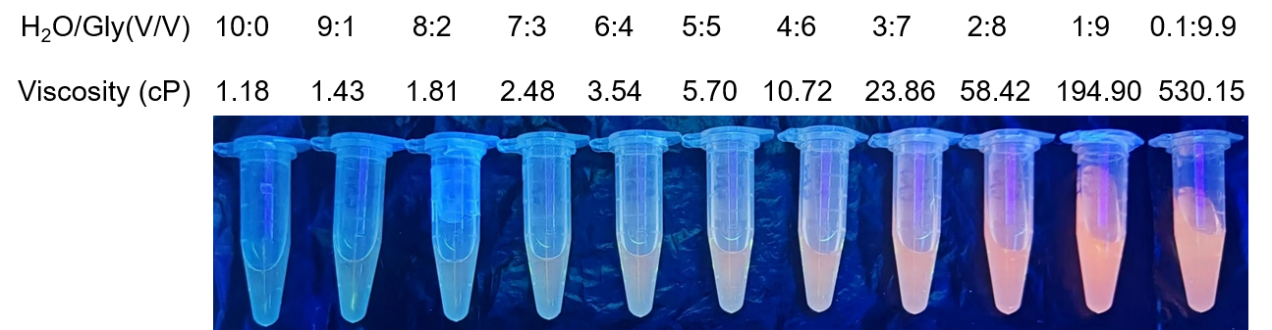


**Figure S10.** Fluorescence image of **DCM-DH** (10 μM) in a water-glycerol system with different viscosities.


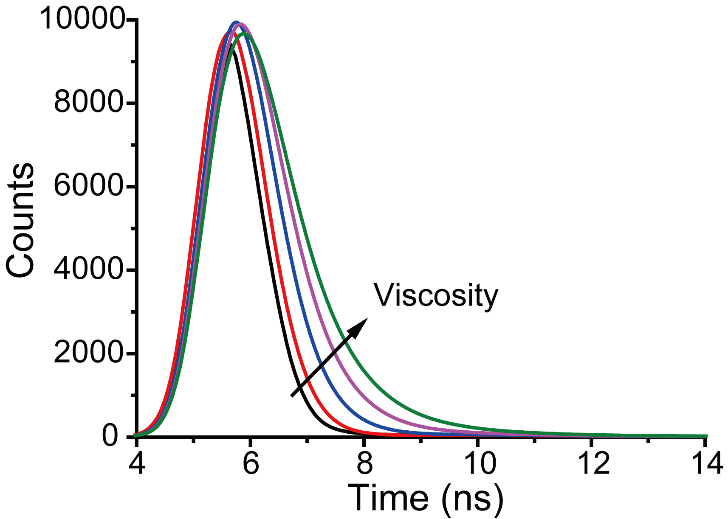


**Figure S11.** Fluorescence lifetime spectra of **DCM-DH** (10 μM) under different viscosities in the water/glycerol solutions.


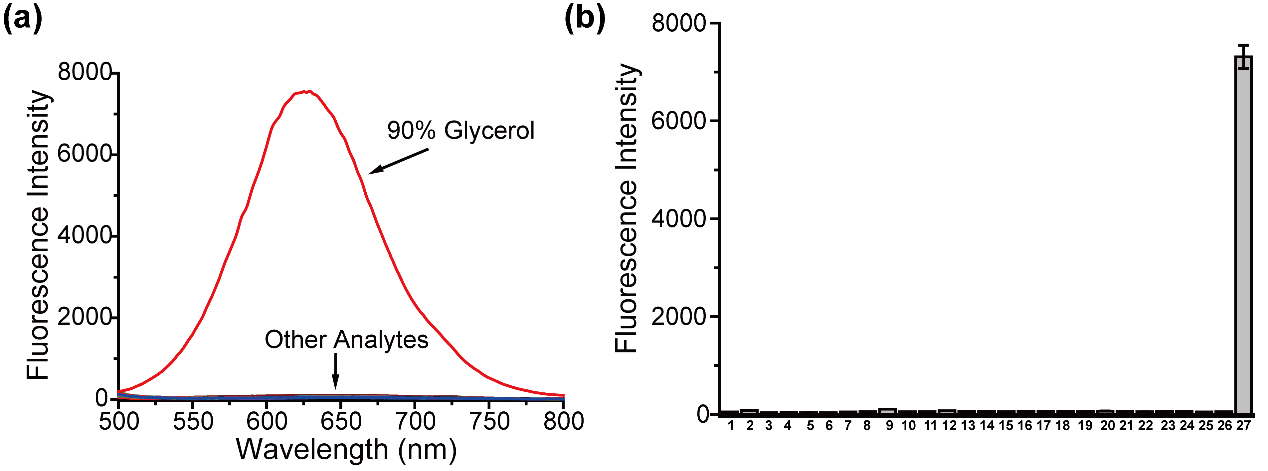


**Figure S12.** (a) Fluorescence spectra and (b) fluorescence intensity of **DCM-DH** (10 μM) to various species. (1) blank; (2) O_2_^• −^ (100 μM); (3) H_2_O_2_ (100 μM); (4) ^1^O_2_ (100 μM); (5) •OH (100 μM); (6) ClO^−^(100 μM); (7) ONOO^−^ (100 μM); (8) CE (100 mU mL^-1^); (9) BSA (100 μg mL^-1^); (10) GOx (100 mU mL^-1^); (11) Catalase (100 mU mL^-1^); (12) Trypsin (100 mU mL^-1^); (13) Glutamine (100 μM); (14) VC (100 μM); (15) Serine (100 μM); (16) Citric acid (100 μM); (17) Glucose (100 μM); (18) Cys (100 μM); (19) Hcy (100 μM); (20) GSH (100 μM); (21) Mg^2+^ (200 μM); (22) Ca^2+^ (200 μM); (23) Fe^3+^ (200 μM); (24) Al^3+^ (200 μM); (25) Cu^2+^ (200 μM); (26) Zn^2+^ (200 μM); (27) 90% Glycerol. λ_ex_/_em_ = 480/640 nm.


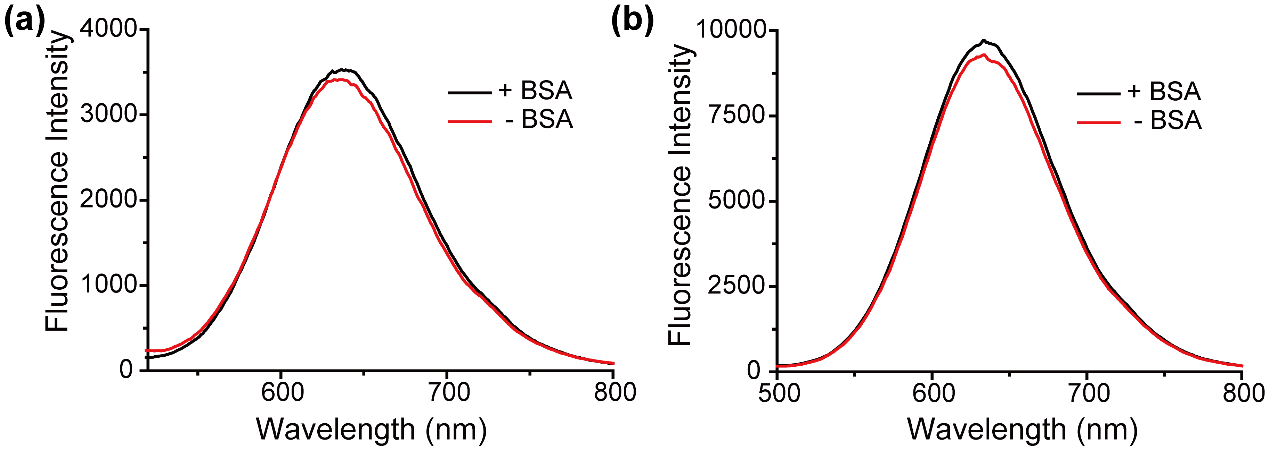


**Figure S13.** Fluorescence spectra of **DCM-DH** (10 μM) and **DCM-DH** (10 μM) + BSA (2 μM) in water/glycerol of different viscosities. (a) 10.72 cP; (b) 58.42 cP. λ_ex_/_em_ = 480/640 nm.


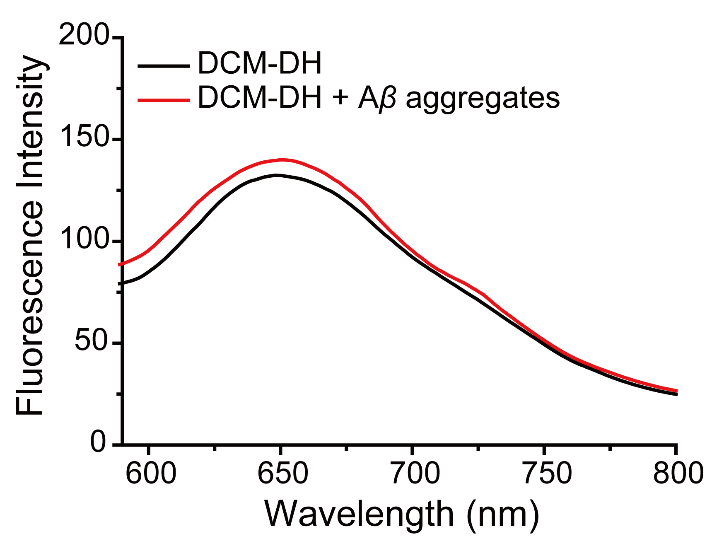


**Figure S14.** Fluorescence emission intensity of **DCM-DH** upon interaction with A*β*42 aggregates (red line) and probes only (black line).


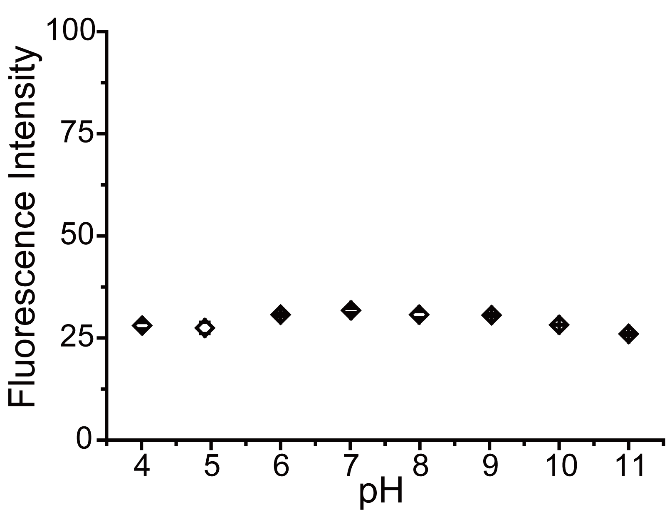


**Figure S15.** Effects of pH on the fluorescence intensity of **DCM-DH** (10 μM) in PBS buffer. λ_ex_/_em_ = 480/640 nm.


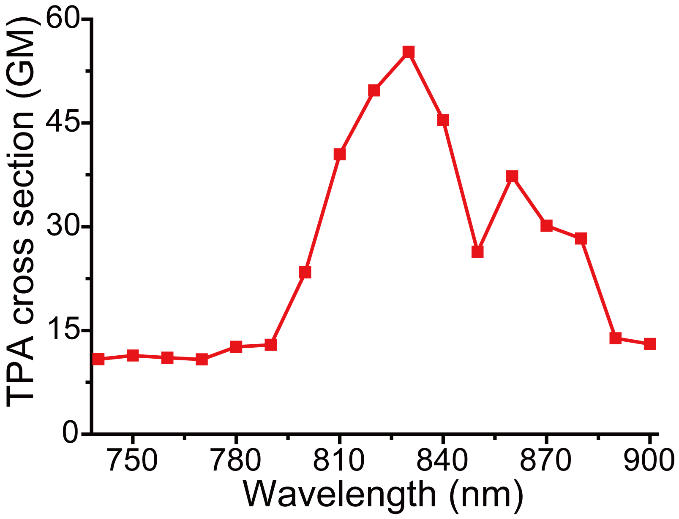


**Figure S16.** The two-photon cross-sections of **DCM-DH** (100 μM) at different wavelength.


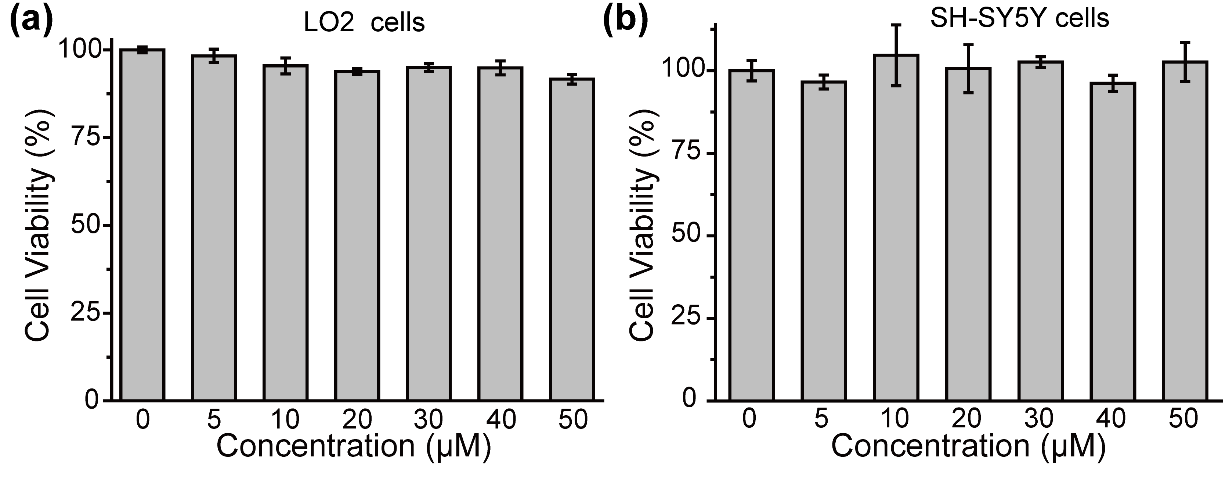


**Figure S17.** The cell cytotoxicity of **DCM-DH**. Cell viability of LO2 cells (a) and SH-SY5Y cells (b) after 6 h of incubation with **DCM-DH** (5, 10, 20, 30, 40, 50 μM). The viability of cells without treatment was defined as 100%. The results were expressed as the mean ± SD (n = 3).


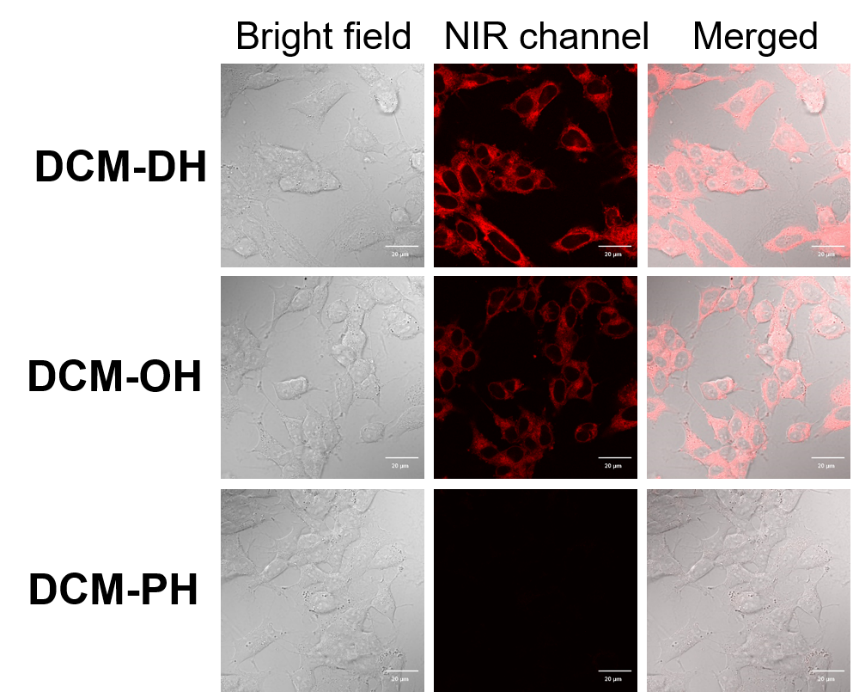


**Figure S18.** Confocal fluorescence imaging of SH-SY5Y cells. Cells were treated with **DCM-DH**, **DCM-OH** and **DCM-PH** (10 μM, λ_ex_ = 488 nm, collected from 600 to 700 nm) for 30 min. Scale bar: 20 μm.


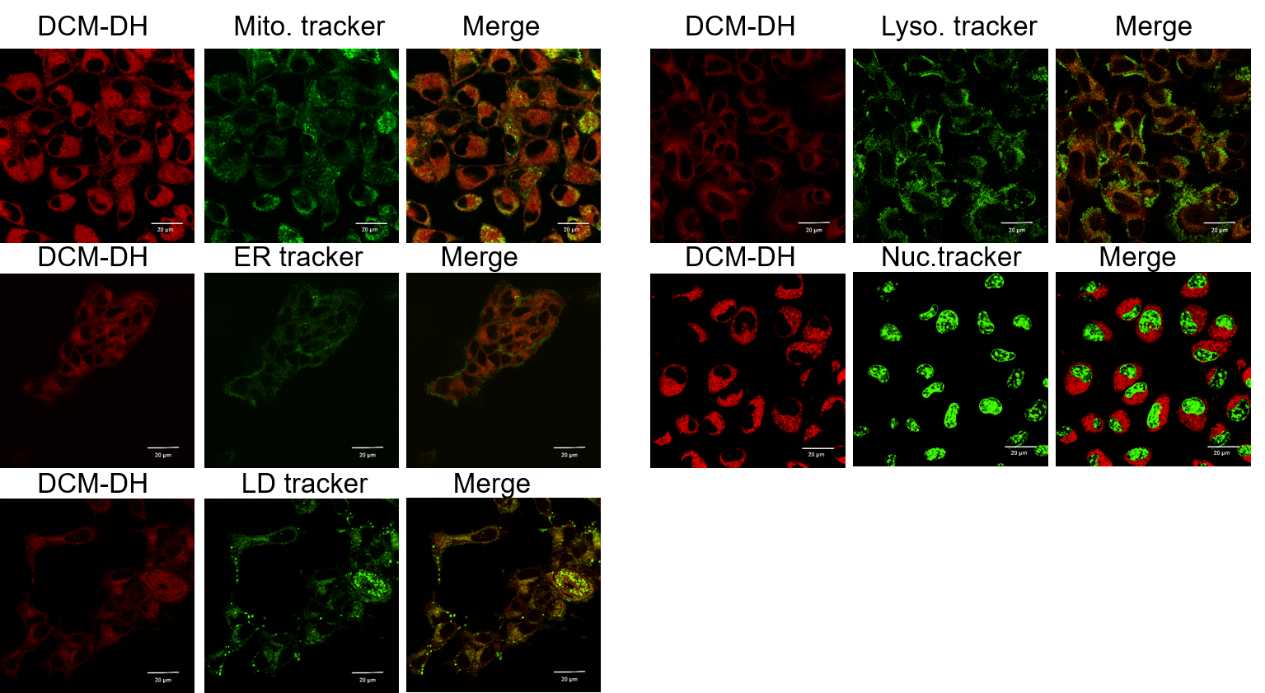


**Figure S19.** Confocal fluorescence images of **DCM-DH** in SH-SY5Y cells. Colocalization images of SH-SY5Y cells co-incubated with **DCM-DH** (10 μM, λ_ex_ = 488 nm, collected from 600 to 700 nm) for the red channel and the corresponding organelle targeting dyes for the green channel (Lyso. tracker, Mito. tracker, ER tracker, Hoechst 33342 for Nuc. tracker, BODIPY for LD tracker). Scale bar: 20 μm.


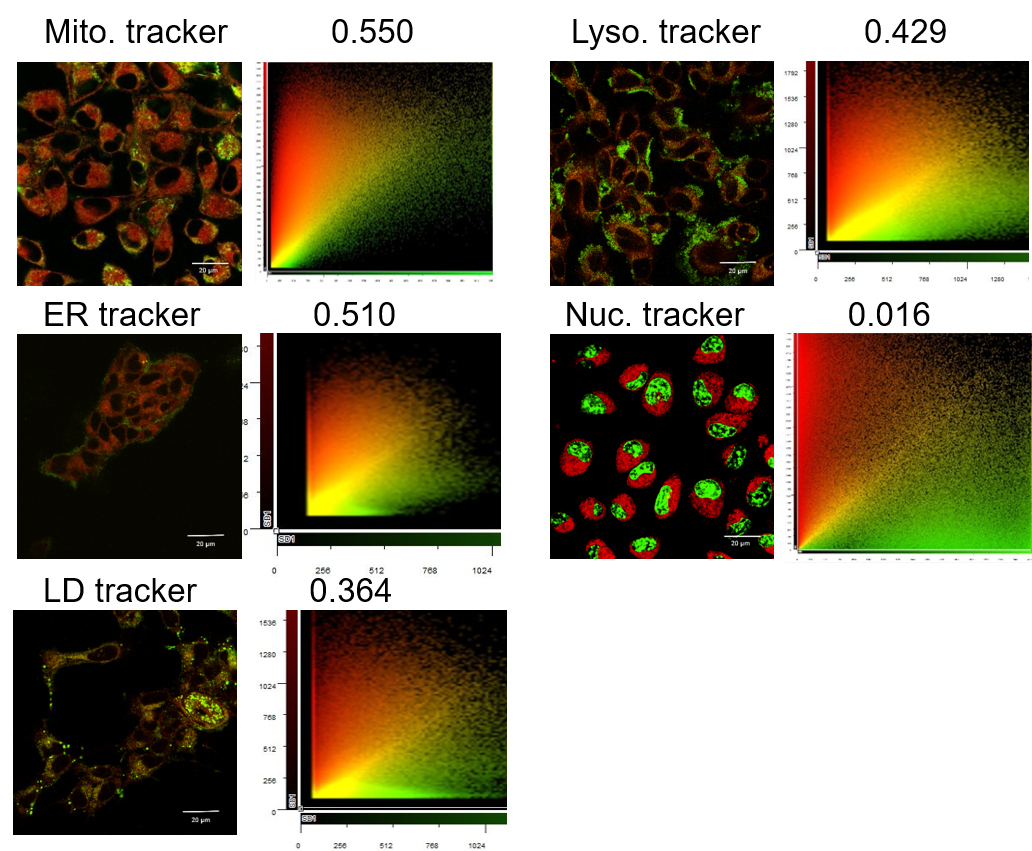


**Figure S20.** Colocalization images of SH-SY5Y cells co-incubated with **DCM-DH** (10 μM, λ_ex_ = 488 nm, collected from 600 to 700 nm) for the red channel and the corresponding organelle targeting dyes for the green channel (Golgi tracker, Lyso. tracker, Mito. tracker, ER tracker, Hoechst 33342 for Nuc tracker, BODIPY for LD tracker). The number on the image is Pearson's coefficient. Scale bar: 20 μm.


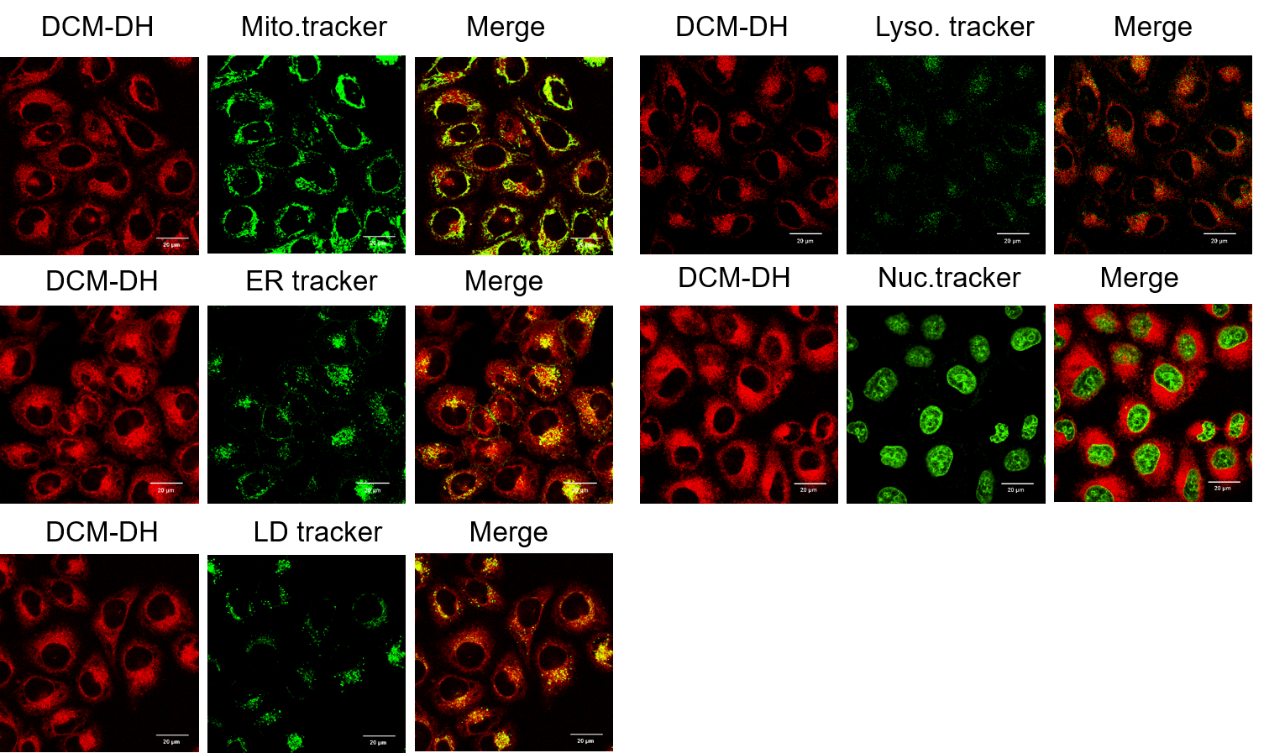


**Figure S21.** Confocal fluorescence images of **DCM-DH** in LO2 cells. Colocalization images of LO2 cells co-incubated with **DCM-DH** (10 μM, λ_ex_ = 488 nm, collected from 600 to 700 nm) for the red channel and the corresponding organelle targeting dyes for the green channel (Golgi tracker, Lyso. tracker, Mito. tracker, ER tracker, Hoechst 33342 for Nuc. tracker, BODIPY for LD tracker). Scale bar: 20 μm.


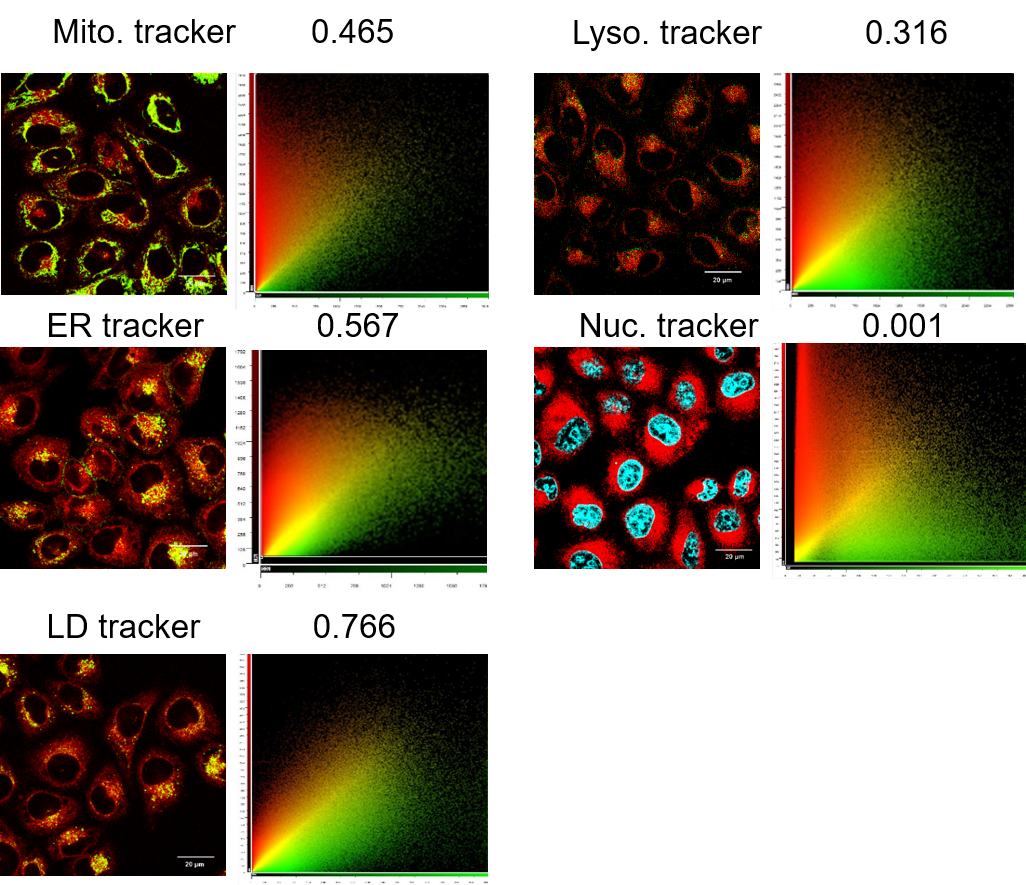


**Figure S22.** Colocalization images of LO2 cells co-incubated with **DCM-DH** (10 μM, λ_ex_ = 488 nm, collected from 600 to 700 nm) for the red channel and the corresponding organelle targeting dyes for the green channel (Golgi tracker, Lyso. tracker, Mito. tracker, ER tracker, Hoechst 33342 for Nuc. tracker, BODIPY for LD tracker). The number on the image is Pearson's coefficient. Scale bar: 20 μm.


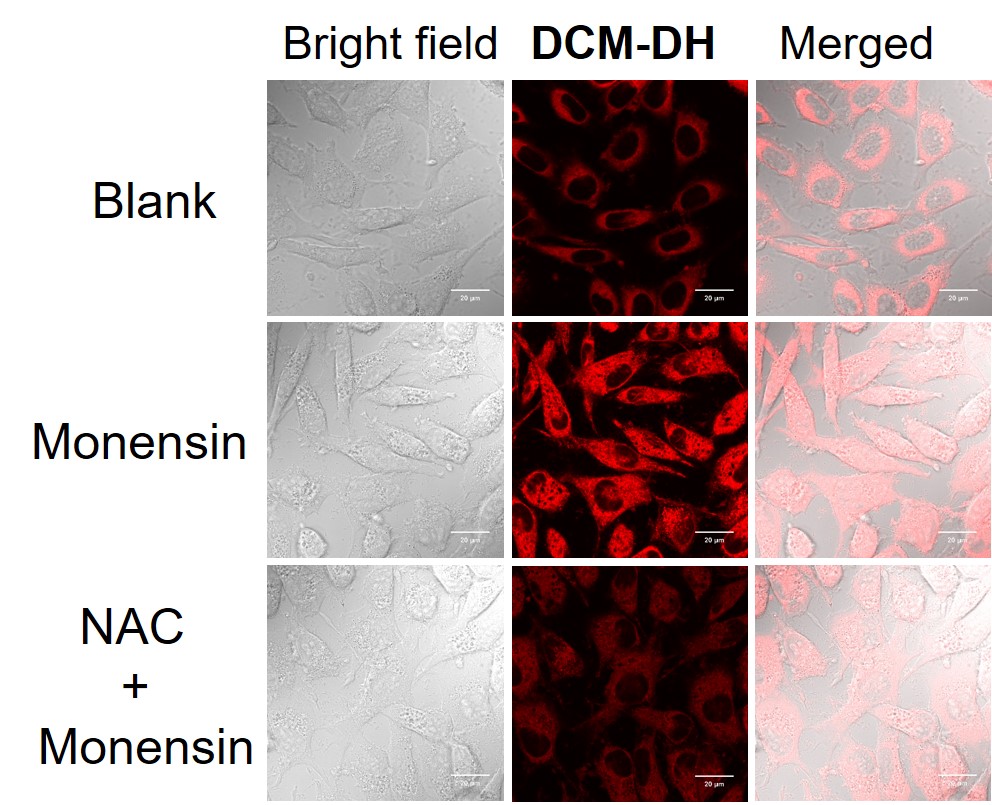


**Figure S23.** Confocal fluorescence imaging of viscosity in SH-SY5Y cells under various treatments. Blank group: cells were incubated with **DCM-DH** (10 μM). The monensin group: cells were pretreated with monensin (10 μM) for 30 min and then incubated with **DCM-DH** (10 μM). The NAC + monensin group: cells were pretreated with NAC (10 μM) + monensin (10 μM) for 30 min and then incubated with **DCM-DH** (10 μM). λ_ex_ = 488 nm, collected 600-700 nm. Scale bar: 20 μm.

**
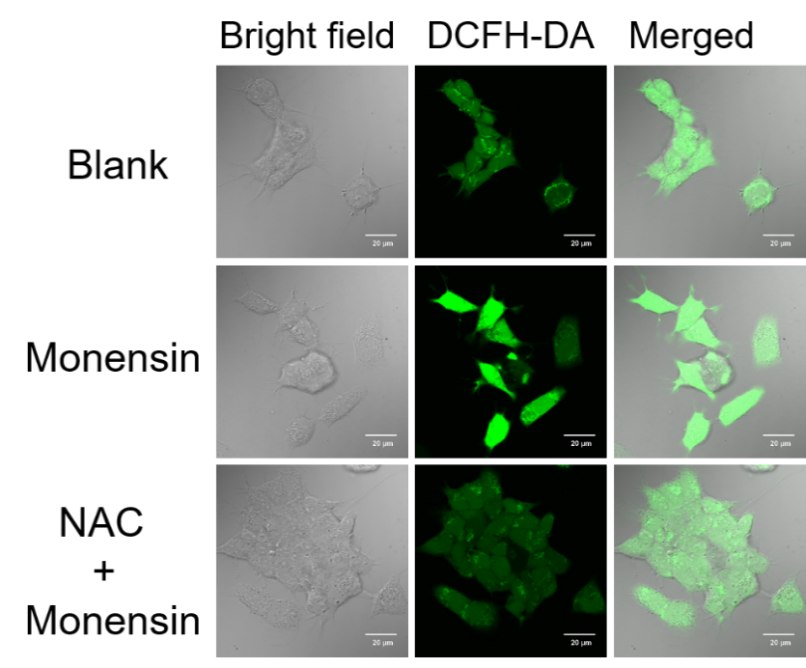
**

**Figure S24.** Confocal fluorescence imaging of viscosity in SH-SY5Y cells under various treatments. Blank group: cells were incubated with DCFH-DA (10 μM). The monensin group: cells were pretreated with monensin (10 μM) for 30 min and then incubated with DCFH-DA (10 μM). The NAC + monensin group: cells were pretreated with NAC (10 μM) + monensin (10 μM) for 30 min and then incubated with DCFH-DA (10 μM). λ_ex_ = 488 nm, collected 500-600 nm. Scale bar: 20 μm.


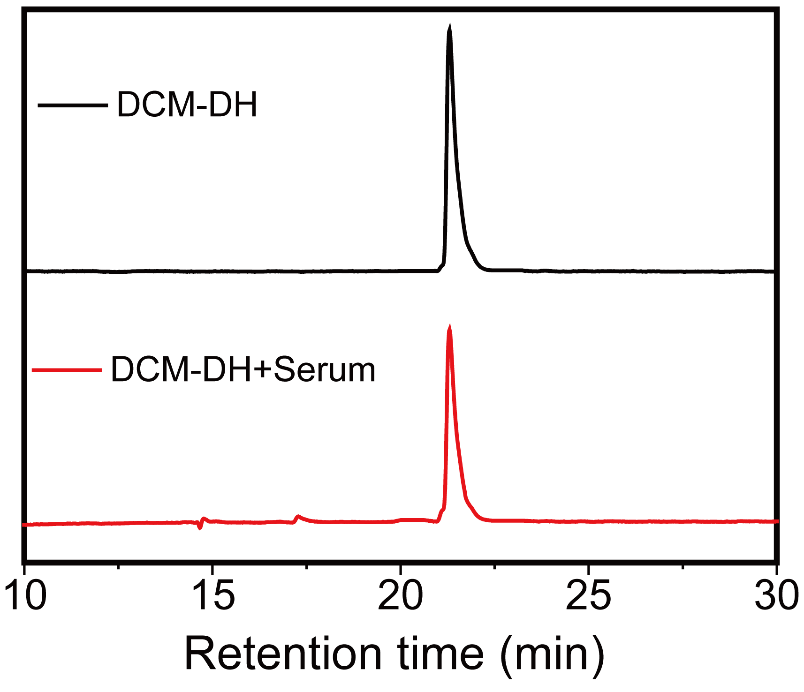


**Figure S25.** *In vitro* stability studies of **DCM-DH** in mouse serum at 37˚C. Chromatograms of **DCM-DH** (1 µM, black line) and serum + **DCM-DH** (1 µM, red line). Gradient: (1) 0 min, 10% B; (2) 30 min, 100% B (all the changes were linear, flow rate: 0.2 mL/min, wavelength: 450 nm).

**
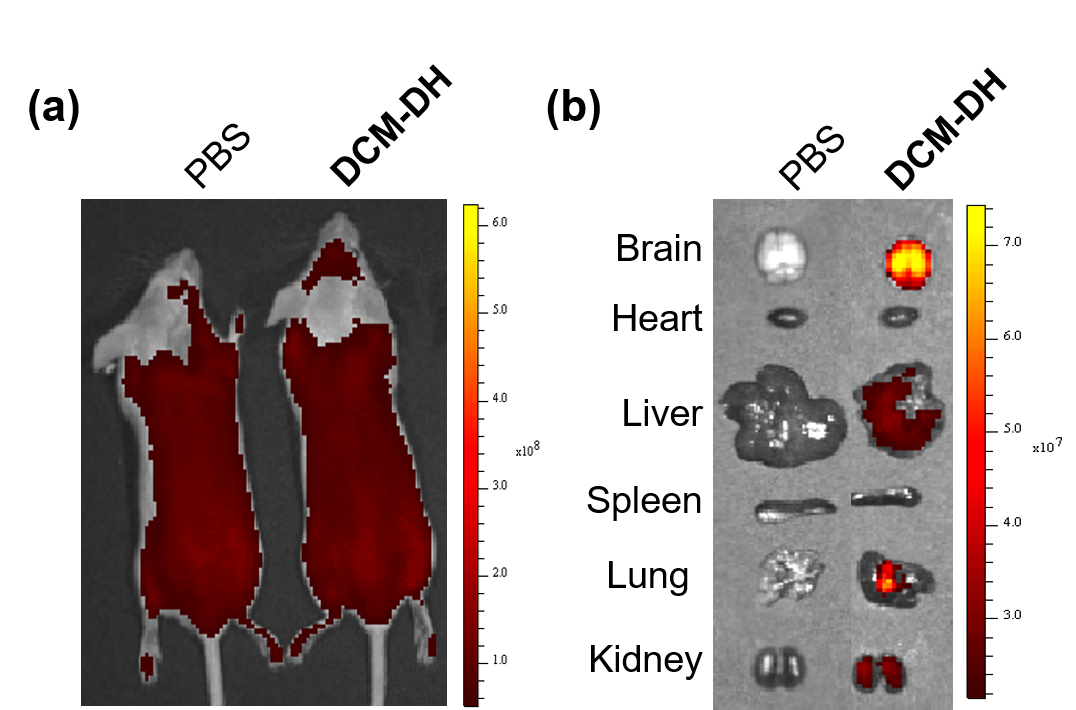
**

**Figure S26.** (a) *In vivo* imaging and (b) biodistribution of **DCM-DH** in BALB/c mice through vein tail injection. Mouse received 0.4 mg kg^-1^ probes and then was euthanized for organ imaging 30 min after injections. The image was obtained under an excitation wavelength of 465 nm and emission filter of 620 nm.

**Table S2**. Comparison of **DCM-DH** with other representative probes for amyloid‑*β* plaques.

| **Probe structure** | **Wavelength**  **λ_ex_, λ_em_** | **Stokes shift** | **Sample type** | **Limitations** | **Reference** |
| --- | --- | --- | --- | --- | --- |
|  | λ_ex_: 480 nm  λ_em_: 640 nm | 160 nm | *In vitro* Brain;  *In vivo* APP/PS1 Tg mice |  | This work |
|  | λ_ex_: 650 nm  λ_em:_:670 nm | 20 nm | *In vitro* Brain sections of APP23 Tg mice;  *In vivo* APP23 Tg mice | Small Stokes shift | Hintersteiner, M. et al.  *Nat. Biotechnol*., 23, 577-583 (2005). |
|  | λ_ex:_ 530 nm  λ_em:_ 585 nm | 55 nm | *In vitro* Brain sections of APP/PS1 Tg mice | Limited emission wavelength;  Large injection dose | Teoh, C. L. et al. *J. Am. Chem. Soc.,* 137,  13503-13509 (2015). |
|  | λ_ex:_ 599 nm  λ_em:_ 678 nm | 79 nm | *In vitro* Brain sections of APP/PS1 Tg mice;  *In vivo* APP/PS1 Tg mice | Large injection dose: 2 mg/kg | Fu, H. et al.  *J. Med. Chem.,* 58, 6972-6983 (2015). |
|  | λ_ex:_ 419 nm  λ_em:_ 566 nm | 147 nm | *In vitro* Brain sections of 5XFAD Tg mice;  *In vivo* Frontal cortex of 5XFAD Tg mice | Limited emission wavelength;  Large injection dose: 5 mg/kg;  Low tissue penetration depth: 172 μm | Shin, J. et al.  *Angew. Chem. Int. Ed. Engl.*, 58, 5648-5652 (2019). |
|  | λ_ex:_ 500 nm  λ_em:_ 550 nm | 50 nm | *In vitro* Brain sections;  *In vivo* APP/PS1 Tg mice | Limited emission wavelength;  Fluorescence intensity increased relatively low (72.82 times, glycerol content from 0% to 90%) | Wang, Y. X. et al. *ACS Appl. Bio Mater.,* 5, 3049-3056(2022). |


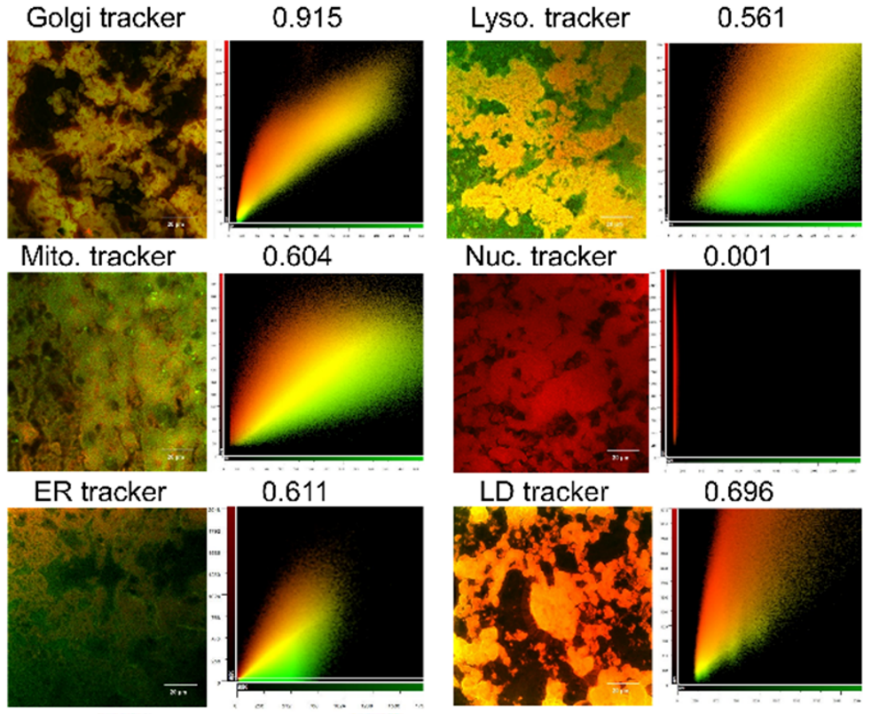


**Figure S27.** Colocalization images of AD mouse brain sections co-incubated with **DCM-DH** and the corresponding organelle targeting dyes. The number on the image is Pearson's coefficient.


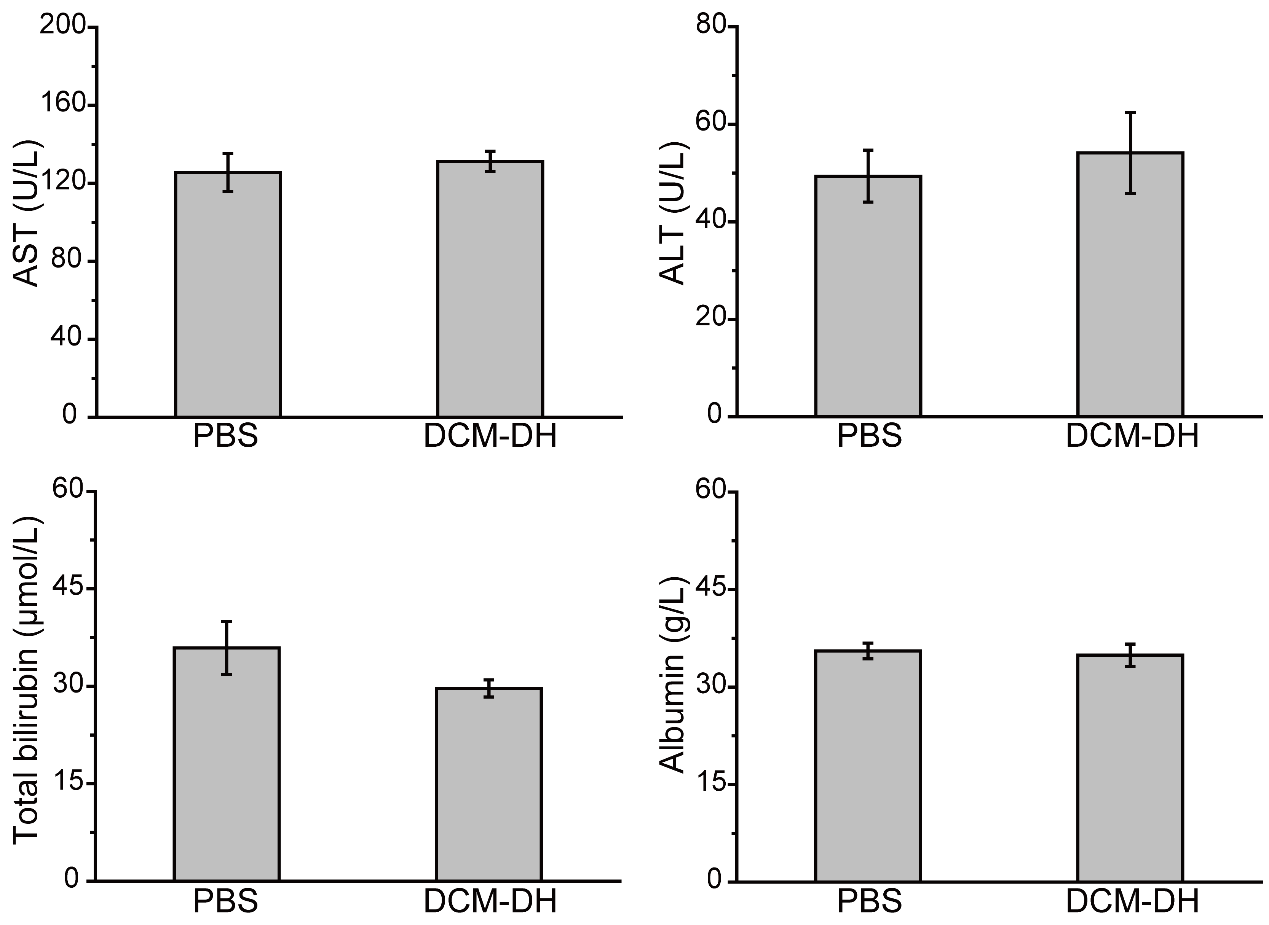


**Figure S28.** Liver function analysis of mice injected with **DCM-DH** as indicated by the change of aspartate transaminase (AST), alanine aminotransferase (ALT), total bilirubin and albumin level in mouse serum. Data represented as mean ± standard deviation (n = 3).


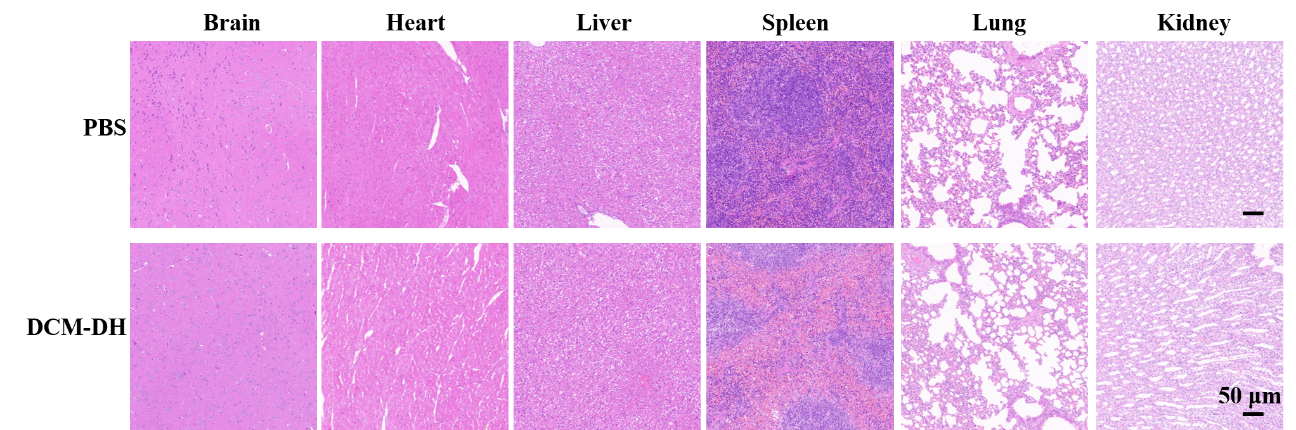


**Figure S29.** H&E analysis of the major organs of BALB/c mice injected with PBS and **DCM-DH** (0.4 mg kg^-1^). Scale bar: 50 μm.

**
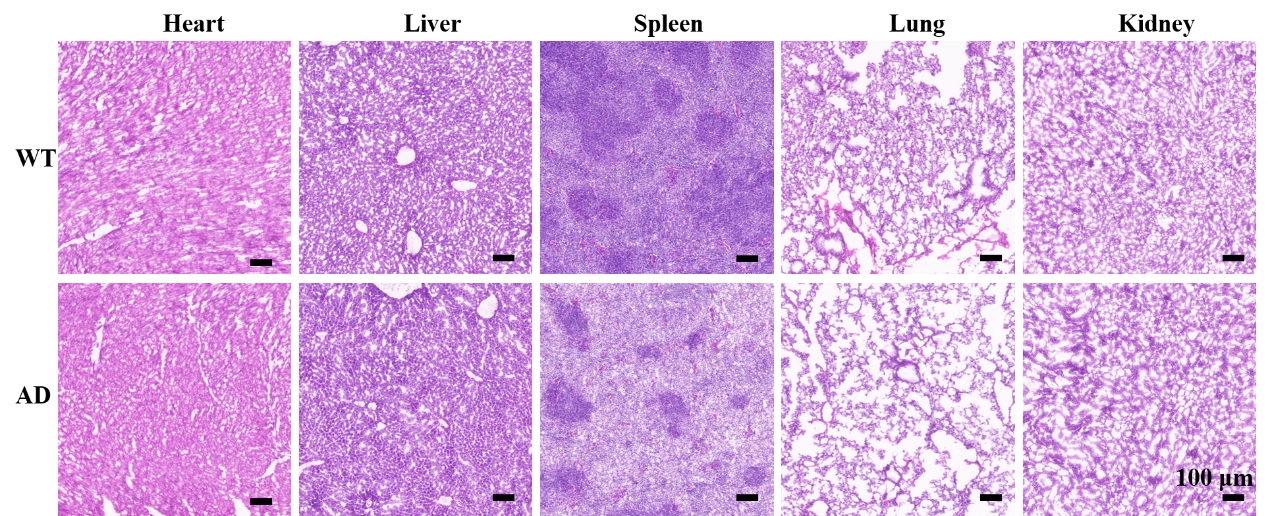
**

**Figure S30.** H&E analysis of the major organs of WT and AD mice injected with **DCM-DH** (0.4 mg kg^-1^). Scale bar: 100 μm.

**
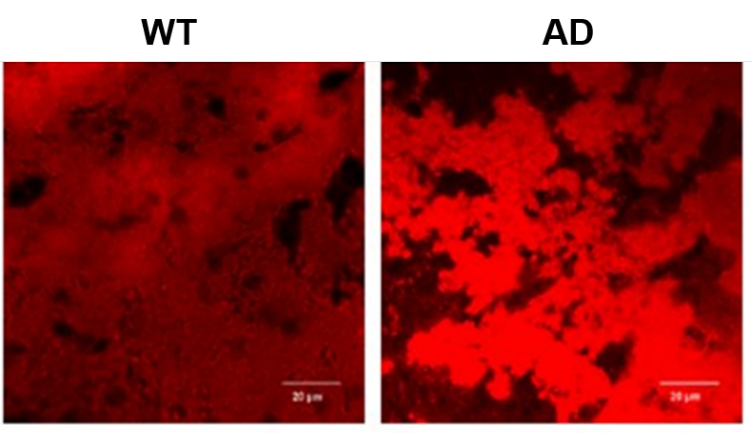
**

**Figure S31.** Confocal images of WT and AD mouse brain sections after **DCM-DH** treatment. Scale bar: 20 μm.

**References**

1. Li, H. *et al*. Ferroptosis accompanied by •OH generation and cytoplasmic viscosity increase revealed via dual-functional fluorescence probe. *J. Am. Chem. Soc.* **141**, 18301-18307 (2019).

2. Wu, W. *et al*. Design and synthesis of a deep tissue penetrating near-infrared two-photon fluorescence probe for the specific detection of NQO1. *Chem. Commun.* **58**, 5634-5637 (2022).

3. Yang, Y. P. *et al*. Developing push-pull hydroxylphenylpolyenylpyridinium chromophores as ratiometric two-photon fluorescent probes for cellular and intravital imaging of mitochondrial NQO1. *Anal. Chem.* **93**, 2385-2393 (2021).

4. Wang, J. et al. Two-photon near infrared fluorescent turn-on probe toward cysteine and its imaging applications. *ACS Sensors* **1**, 882-887 (2016).

5. Wu, X. et al. Near-infrared two-photon fluorogenic probe for imaging orthotopic hepatocellular carcinoma chemotherapy. *Angew. Chem. Int. Ed.* **60**, 15418-15425 (2021).
